# Three-Dimensional Packed-Bed Electrochemical Reactor Design for Selective Selenite Reduction in Water

**DOI:** 10.1021/acsestengg.5c00861

**Published:** 2025-12-29

**Authors:** Zilan Yang, D. Ricardo Martinez-Vargas, Ao Xie, Shengcun Ma, Shiqiang Zou

**Affiliations:** † Department of Civil and Environmental Engineering, 3078 Michigan State University, East Lansing, Michigan 48824, United States; ‡ Energy Storage and Distributed Resources Division, 1666 Lawrence Berkeley National Laboratory, Berkeley, California 94720, United States

**Keywords:** selenium removal, three-dimensional electrochemical reactor, particle electrodes, flue-gas desulfurization wastewater, electrochemical reduction

## Abstract

Selenium (Se) contamination in flue-gas desulfurization (FGD) wastewater from coal-fired power plants poses significant environmental and regulatory challenges. Here, we developed and optimized a three-dimensional electrochemical reactor (3DER) with carbon-based particle electrodes (PEs) to remove Se­(IV). Compared with conventional two-dimensional systems, the 3DER provides an enlarged electrode surface area, enabling faster removal kinetics and higher resilience without regeneration. Reactor performance was systematically evaluated as a function of PE geometry, recirculation rate, cell potential, and anode-to-cathode (A:C) chamber ratio. The optimized configuration (A:C = 1:2, *E*_cell_ = –2.1 V, recirculation rate 3.3 mL min^–1^) balanced cathodic efficiency while minimizing anodic parasitic reactions. In synthetic wastewater containing 0.1 mM Se­(IV), the single-pass 3DER achieved steadily increasing performance, with hourly removal improving from 61.3% in the first hour to 68.1% by the 12th hour. Applied to real FGD wastewater, the system maintained an average hourly removal of 51.7% (4.2 mg of Se L^–1^ h^–1^) without regeneration and reached a specific energy consumption as low as 0.03 kWh g^–1^ Se despite high chloride levels. Competing ions, including Mn and Si, further enhanced the Se reduction by forming oxide layers and rejecting Cl^–^ from the electrode surface. Enhanced kinetics under elevated Se­(IV) loadings yielded a peak removal of 74.4% (17.5 mg of Se L^–1^ h^–1^). These results demonstrate robust and efficient removal performance of the 3DER, supporting its promise for selenium-rich wastewater treatment and future scale-up.

## Graphical abstract

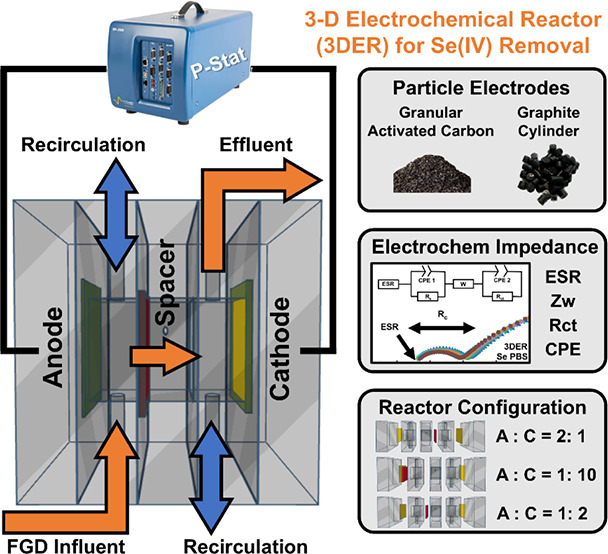

## Introduction

Selenium (Se) is an essential trace element for humans and has been widely used to produce commercial products such as semiconductors and fertilizers.^1–3^ Human activities have significantly increased the amount of Se released into the environment.^4,5^ One major pathway of anthropogenic Se release is through the flue gas desulfurization (FGD) wastewater generated from coal-fired power plants, which often contains inorganic Se­(IV) and Se­(VI) oxyanions.^6,7^ The U.S. Environmental Protection Agency (EPA) has enacted a regulation to have a discharge limit of 29 μg L^–1^ Se daily maximum in FGD effluent, while some other Se-impacted water matrices, such as agricultural drainage, do not have specific Se guidelines.^8^ Instead, agricultural practices focus on buffer and barrier installation to sequester and separate Se.^9–11^ Although Se­(VI) is more prevalent in the aforementioned wastewaters, Se­(IV) is reportedly more mobile and toxic.^12–14^ Excessive Se discharge could accumulate in the food chain and result in health concerns, even cancer.^1,15^ Removing dissolved Se­(IV) from impacted water matrices is therefore urged to safeguard public health.

Existing technologies for removing dissolved Se­(IV) include biological, chemical, and electrochemical treatments.^16–18^ Among all treatment technologies, Se removal via direct electrochemical reduction (SeDER) has been found to offer several unique advantages, such as negligible solid generation, minimum chemical addition, and reduced risk associated with the generation of more bioavailable organo-Se species.^17,19^ The SeDER technology primarily targets FGD wastewater characterized by elevated water temperatures, as the operation relies on continuous electroplating of conductive Se(0) at a temperature ≥75 °C.^20^ The SeDER system achieved an average removal efficiency of 94% for 0.1 mM Se­(IV) in a synthetic water matrix, with no chemical addition, negligible waste production, and minimal secondary pollution.^21^ Despite its high removal efficiency, the kinetics of SeDER were constrained by the unoptimized batch operation mode and the limited surface area of the planar electrode. Further, wastewater with ambient temperature and lower Se­(IV) concentrations, such as agricultural drainage, may present significant operation constraints in 4-electron Se­(IV)/Se(0) electroplating.

In contrast to the 4-electron pathway, the 6-electron pathway converts Se­(IV) to Se­(−II). The generated Se­(−II) further reduces the remaining Se­(IV) to suspended Se(0) particles. This pathway enables continuous Se­(IV) removal at ambient temperature, as the Se(0) particles remain suspended instead of being plated onto the electrode surface. Better engineering designs can further resolve the mass transfer constraints. For example, recent studies have investigated three-dimensional electrochemical reactors (3DERs) for their abundant surface area and fast reaction kinetics when treating diverse wastewaters, such as coke wastewater, dye wastewater, and landfill leachate.^22–24^ The 3DER utilizes conductive particle electrodes (PEs) to fill the reactor, employing various PE materials made of C (e.g., graphite and granular activated carbons), Ni, Co, and other metals.^22–25^ These PEs extend the primary anode or cathode to minimize the transport distance between targeted pollutants and an electrochemically active surface.^26^ To our knowledge, most reported 3DER systems operate in batch mode to oxidize organic pollutants or reduce chromate and nitrate, which yield soluble end products.^27,28^ However, there have been limited 3DER initiatives to reduce metal and metalloid pollutants, where the solid-phase reduction products such as Se(0) can accumulate in the reactor, occupy the reaction sites, and shorten the electrode lifespan.^29^ Moreover, when more negative potentials are applied to enhance Se­(IV) removal, the emergence of competing reactions (e.g., water-splitting reactions) can lead to significant performance degradation over time. A slower flow rate offers an extended hydraulic retention time, but it may be insufficient to prevent possible Se(0) precipitation and gas bubble adherence on the electrode surface. In addition to balancing reaction kinetics and reactor hydraulics, evaluating a 3DER system in complex wastewaters is equally important, where competing anion concentrations may exceed Se­(IV) levels by up to 4 orders of magnitude.^7,17^

In this article, we developed a 3DER reactor to treat Se­(IV)-laden wastewater, aiming to assess electrochemical removal performance and refine the reaction kinetics and hydraulics to balance Se(0) generation and discharge. The specific objectives of this study are to (1) quantify the Se­(IV) removal kinetics using two selected PE materials, (2) evaluate the impact of operating parameters, such as flow rate, applied potential, and spacer placement, on electrochemical Se­(IV) removal, (3) determine the influence of competing ion concentrations on key performance metrics, (4) regenerate PE materials to cleanse Se(0) deposits and restore reaction kinetics, and (5) explore 3DER performance under single-pass operation, treating synthetic and real FGD wastewaters. We aim to develop a scalable engineering design and an optimized operational protocol for the 3DER system, ensuring consistent and efficient treatment of Se-impacted wastewater.

## Materials and Methods

### Experimental Setup

The electrochemical reactor was constructed from 5 acrylic plates. The two end plates have an external dimension of 10 × 10 × 1 cm, while the three middle plates have an external dimension of 10 × 10 × 1.5 cm. The three middle plates were cut in the center, resulting in an internal chamber dimension of 5 × 5 × 1.5 cm for each plate. Rubber gaskets (Danco, 10 × 10 × 0.1 cm) were cut to size and placed between the acrylic plates to prevent leakage. In the 2D electrochemical reactor (2DER) setup, two identical planar graphite foils (Fisher Scientific, 0.254 mm thick, 99.8% metal basis) served as the anode and cathode with a total electrode area of 50 cm^2^. This reactor could transition from a 2D to a 3D electrochemical reactor by incorporating PEs as filling materials within the internal chamber (SI, Figure S1). These PEs included GAC (EnviroSupply, 4 × 8 mesh-sized coconut shell carbon) with a specific surface area of 1091.7 m^2^ g^–1^, or graphite cylinders (GC) with a specific surface area of 3.0 cm^2^ g^–1^ (Dingjingxin Industry, Ø7 × 5 mm). Accordingly, 3D reactors were denoted as 3DER-GAC or 3DER-GC. In the 3D reactor setup, a nonconductive perforated nylon spacer (Caydo, 6 × 6 × 0.1 cm with 7 holes per cm^2^) was cut to size and placed at 0, 1.7, or 3.3 cm apart from the planar graphite anode to separate the reactor into a cathodic chamber and an anodic chamber. The spacer separates PEs to prevent system short circuits and allows tuning of the working electrode area and potential. The fully assembled reactor, with acrylic plates, rubber gaskets, and a perforated spacer, has a measured working volume of 120 mL (SI, Figure S1). Due to the presence of PEs, neither mechanical nor magnetic stirring was provided in any of the 2D and 3D reactors.

### Operating Modes

Prior to testing, electrochemical reactors were filled with blank 100-mM KH_2_PO_4_ phosphate-buffered saline (PBS) solution and left overnight for wetting. There is no recirculation or working potential applied to the system, and the open circuit voltage (OCV) is measured to be in the range of 0–0.2 V. The wetting process improves electrolyte contact with the carbon-based PEs, as the nonpolar carbon surfaces are inherently hydrophobic.^30^ This blank PBS solution was fully drained before testing. In static operating mode, the 2DER was filled with 120 mL of blank PBS or synthetic wastewater (PBS with 0.1 mM Na_2_SeO_3_, unless otherwise stated), while the 3DER was filled with 50 mL due to the internal chamber space occupied by PEs. All solutions were adjusted to pH = 5.5 to simulate FGD wastewater.^19^ In recirculation operating mode, a total of 200 mL of synthetic wastewater was circulated between a 2D or 3DER and an external reservoir using a peristaltic pump (Masterflex L/S MasterSense, VWR). When needed, a defined concentration of NaCl, NaNO_3_, or Na_2_SO_4_ was spiked into the synthetic wastewater. A magnetic stirrer was placed in the external reservoir to thoroughly mix the solution at 200 rpm. In single-pass operating mode, the 3DER-GC was continuously fed with either synthetic or real FGD wastewater (SI, Table S1), using flow rates and cell voltage established in the recirculation mode. The electrochemical reactor was connected to a BioLogic SP-300 potentiostat under a two-electrode configuration with varying cell potentials exerted across the working and counter electrodes. An additional channel of BioLogic SP-300 was used to monitor the potential of working and counter electrodes, referenced against a leakless miniature Ag/AgCl electrode (3.5 M KCl, eDAQ, Model ET072). The Ag/AgCl reference electrode is placed 2.5 cm away from the graphite sheet cathode (SI, Figure S1). All chemicals were purchased from VWR and used directly without further purification (purity >99.9%). DI water from the Milli-Q system was used to prepare solutions and clean glassware.

### Experimental Procedure

All experiments were conducted in duplicate at room temperature (25 ± 2 °C). Initially, we ran reactors in static mode to establish the performance baseline of 2DER, 3DER-GAC, and 3DER-GC. This phase of the experiments began with physical adsorption tests in all reactors fed with synthetic wastewater to evaluate their Se­(IV) removal capacity through carbon adsorption. We then used the potentiostat to maintain a cell potential (*E*_cell_) of −1.9 V, resulting in a stabilized working electrode potential (*E*_we_, cathode) of −0.7 V (vs Ag/AgCl, unless otherwise stated) and a counter electrode potential (*E*_ce_, anode) of 1.2 V (SI, Figure S3). Previous studies reported an *E*_we_ of −0.6 V can facilitate the electrochemical Se­(IV) reduction through a 6-electron Se­(IV)/Se­(−II) pathway in a 2D system.^19,21^ We validated the potential in 3DER by performing cyclic voltammetry (CV) scans with 3DER and confirmed that −0.7 V is the minimum *E*_we_ for Se­(IV) reduction (Supporting Information, Figure S2). All tests in static mode lasted 3 h, and no stirring was provided within the reactor. Water samples were collected at the onset and at the conclusion of each test. We thoroughly cleaned the reactor with DI water and filled it with pristine PEs between tests.

Next, we investigate the dynamics of electrochemical impedance with regard to the operation parameters, such as *E*_cell_ and flow rate. Experiments were conducted with 0.1 mM Se­(IV) synthetic wastewater under recirculation modes. The reactor was operated for 3 h at an *E*_cell_ of −1.9, −2.1, and −2.3 V, and flow rates were set to 0.8, 1.7, and 3.3 mL min^–1^ for each *E*_cell_. The water sample was collected at the onset and conclusion of the experiments, and the corresponding electrochemical impedance spectroscopy (EIS) measurement was taken immediately after the 3-h operation. At the conclusion of each experiment, the used PEs were thoroughly rinsed with DI water, soaked in DI water overnight, and sonicated in DI water for 5 min before the next test. This PE cleaning protocol is consistently applied for subsequent tests unless otherwise specified. The protocol is validated with both experimental and SEM results (Supporting Information, Figure S2). Water samples were collected from the well-mixed external reservoir at the onset and conclusion of each experiment.

We then evaluated the influence of the nylon spacer placement on the electric field distribution and Se­(IV) removal kinetics. Besides the initial setup with only one parallel spacer placed 2 cm from the cathode, we tested two additional setups with one parallel spacer placed 0 and 3 cm from the cathode. These experiments are designed to investigate the effect of the anode-to-cathode chamber ratio and, consequently, the anode-to-cathode electrode effective surface area ratio on reactor performance. The setup and assumption for the potential distribution simulation are included in Supporting Information, Section 8. The same cleaning and water sampling protocols were applied in this part of the experiment.

After optimizing the reactor configuration and operation parameters, we tested the 3DER-GC in single-pass mode to evaluate its performance in continuous operation, providing insights into PE regeneration or replacement frequency. Synthetic wastewater with 0.1 mM Se­(IV) was used for the baseline test. The single-pass flow rate was set at 1.1 mL min^–1^ to match the treatment rate of the prior recirculation tests (200 mL treated in 3 h). We added recirculation at 3.3 mL min^–1^ to enhance the mass transfer within the reactor, as established during the previous tests. Each cycle lasted 12 h, with water samples collected every hour for the first 6 h and at the 12th hour. We then switched to real FGD wastewater collected from a local coal-fired power plant (CFPP) for the 12-h single-pass experiment. FGD wastewater A (FGD-A) was collected after the initial hydrocyclone process at the CFPP, and the wastewater contains high concentrations of background ions and suspended solids (SI, Table S1). The FGD-A was filtered through a 0.45 μm membrane to remove suspended solids prior to experimentation; no pH adjustment was provided, and the measured pH is at 6.3 ± 0.1. FGD wastewater B (FGD-B) was collected after the sand filtration process at the CFPP, with a more diluted background ion concentration and negligible suspended solids. FGD-B was used directly in experiments without any filtration or pH adjustments, with a pH of 6.5 ± 0.1. Both hydrocyclone and sand filtration processes are part of the power plant’s existing treatment train (SI, Figure S2). Both FGD-A and FGD-B were spiked with 0.1 mM Se­(IV) to maintain initial Se­(IV) levels comparable with the synthetic wastewater, as the real FGD wastewater samples we received had inconsistent Se­(IV) concentrations. Se levels in FGD wastewater vary widely among coal-fired power plants due to differences in coal sources and combustion conditions.^31^ The selected spiking concentration (0.1 mM Se­(IV)) falls within the typical range reported and enables consistent performance comparison across all matrices.^32^ Between each 12-h single-pass test, we thoroughly washed the 3DER-GC with DI water and refilled it with cleaned PEs prepared using the previously described cleaning protocol.

### Analytical Methods

Electrical current, electrode/cell potential (*E*_we_, *E*_ce_, and *E*_cell_), chronoamperometry (CA), electrochemical impedance spectroscopy (EIS), and energy consumption data were recorded with the BioLogic potentiostat and EC-lab software.^33^ We conducted EIS at the OCV and an *E*_cell_ of −1.9, −2.1, and −2.3 V with a scan range of 10^5^ to 10^–2^ Hz, a potential amplitude of 10 mV, and 10 points per decade. The EIS result was plotted in the Nyquist format to analyze the change in equivalent series resistance (ESR) and the impact of *E*_cell_ and flow rate on interparticle charge transfer resistance (*R*_ct_) and Warburg coefficient (σ, Ω s^–1/2^). ESR, *R*_ct_, and σ are obtained through an EC-lab fitted equivalent circuit. The squared σ value is inversely related to the diffusion coefficient and is used to measure the diffusion effectiveness in the electrochemical reactor.^34^

The collected water samples were filtered through a 0.45 μm PTFE syringe filter (VWR) and stored in a 4 °C refrigerator before water quality analysis. The total soluble Se concentration was quantified by using an iCAP-RQ inductively coupled plasma mass spectrometer (ICP-MS, Thermo Fisher Scientific) using the Standard Method 3125-B.^35^ The used PEs were collected, rinsed with DI water, and then dried in a fume hood before being analyzed for surface morphology and elemental composition using a Zeiss EVO50 Scanning Electron Microscopy (SEM, ZEISS) and an Oxford INCA energy-dispersive X-ray spectroscopy (Oxford Instruments). Se(0) particle size distribution was measured using a Litesizer 500 Particle Analyzer (Anton Paar). The surface area and pore volume distribution of the GAC were determined by N_2_ physisorption analysis at 77 K using a TriStar II Plus High Throughput Surface Area and Porosity Analyzer (Micromeritics Instrument). The point of zero charge (pH_pzc_) was determined by potentiometric mass titrations with detailed steps in Supporting Information (SI, Table S2).^36,37^ A Fourier-transform infrared spectroscopy (FTIR) study was conducted to study the chemical characteristics of GAC PEs. The spectra were collected using a Nicolet iS 10 FTIR system (Thermo Fisher Scientific) in the range of 400 to 4000 cm^–1^. All spectra were acquired in transmission mode using the KBr disc method.^38^

Se removal efficiency (*E*, %, eq 1) is determined using the initial (*C*_i_, mg L^–1^) and final (*C*_f_, mg L^–1^) total soluble Se concentrations, assuming that there is no significant change in water volume during each test:

1 $$E=\left(C_{i}-C_{f}\right)/C_{i}\times 100\%$$

The specific Se removal rate (*R*, mg L^–1^ h^–1^, eq 2) is calculated over a specified operating period (*t*, h), assuming a constant treated water volume:

2 $$R=\left(C_{i}-C_{f}\right)/t$$

The ratio of reactive surface area to the reactor’s working volume (*V*_R_, mL) was calculated using eq 3 (RA/RV, m^2^ cm^–3^). For the 2DER, the reactive surface area includes only the exposed sides of two submerged graphite foil electrodes (*S*_GF_, m^2^). For 3DER, the reactive surface area also includes the contribution from the filled PEs, estimated by multiplying the average specific surface area of GAC or GC PEs (*S*_PE_, m^2^ g^–1^) by their corresponding mass (*m*, g):

3 $$RA/RV=S_{GF}/V_{R}\;\mathrm{or}\;(S_{GF}+S_{PE}\times m)/V_{R}$$

Specific energy consumption (SEC, kWh g^–1^ Se) was quantified via eq 4, based on the electrical power (*P*, kW) consumed and recorded by the potentiostat and treated water volume (*V*_w_, L):

4 $$\mathrm{SEC}=P\times t/\left(\left(C_{i}-C_{f}\right)\times 1g/1000\mathrm{mg}\times V_{w}\right)$$

## Results and Discussion

### Performance Baseline of 3DER

When operated in an open circuit, the 2D and 3DER exhibited distinct adsorption performances for Se­(IV) oxyanions, influenced by the RA/RV ratio, physisorption through van der Waals interactions plus electrostatic attractions, as well as chemisorption via hydrogen, covalent, and ionic bonding.^39^ A lower RA/RV ratio typically results in fewer bonding active sites and a reduced likelihood of adsorbate interaction with the adsorbent surface. For instance, 2DER showed 0% adsorption over 3 h due to a low RA/RV ratio of 4.2 × 10^–5^ m^2^ cm^–3^ (Figure 1A). Although 3DER-GC had a higher RA/RV ratio of 2.5 × 10^–4^ m^2^ cm^–3^, it also exhibited 0% adsorption. This was due to GC’s neutral pH_pzc_ of 7.4 and its weakly positively charged surface at a solution pH of 5.5, leading to minimal electrostatic attraction of negatively charged Se­(IV) oxyanions (Figure 1B). In contrast, 3DER-GAC achieved 63.1% Se­(IV) adsorption in 3 h. BET analysis revealed that GAC was predominantly microporous, with 99.3% of its pores smaller than 2 nm, contributing to a large RA/RV ratio of 363.9 m^2^ cm^–3^ and facilitating physical adsorption. FTIR analysis detected O–H (3250 cm^–1^), C–H (2760 cm^–1^), and C=O (1670 cm^–1^) functional groups on the GAC surface (Figure 1C).^40–42^ The presence of oxygenated surface functionalities (O–H and C=O) indicates enhanced polarity and potential hydrogen-bonding capability, thereby facilitating the adsorption of Se­(IV) oxyanions primarily via hydrogen bonding (O–H).^43–45^ The spectrum also shows features that could correspond to basic functional groups such as C=C (1547 cm^–1^), C=N (pyridinic, 1570 cm^–1^), and N–H (amine/amide, 3359 cm^–1^) on the GAC surface, with some peaks overlapping with those of the oxygenated groups.^46–48^ These basic functionalities may act as electron-donating (C=C) or proton-accepting sites (C=N and amine-type N–H), modulating the local electronic environment and surface polarity, which may favor Se­(IV) adsorption. In addition, GAC had a much higher pH_pzc_ of 10.7, compared to GC (pH_pzc_ = 7.4), to favor the electrostatic attraction of Se­(IV) oxyanions in a solution pH of 5.5 (Figure 1B).^36^ All these factors contributed to rapid initial adsorption at 4.17 mg L^–1^ h^–1^, which diminished to 0.61 mg L^–1^ h^–1^ by the third hour due to decreased Se­(IV) concentration in the solution and occupied GAC adsorption sites.^49^ While GAC shows promising Se­(IV) removal in open circuit operations, its adsorption capacity is limited, nonselective, and highly reversible, leading to inconsistent Se­(IV) removal when treating real wastewater with complex compositions.^50–52^

**Figure 1:**
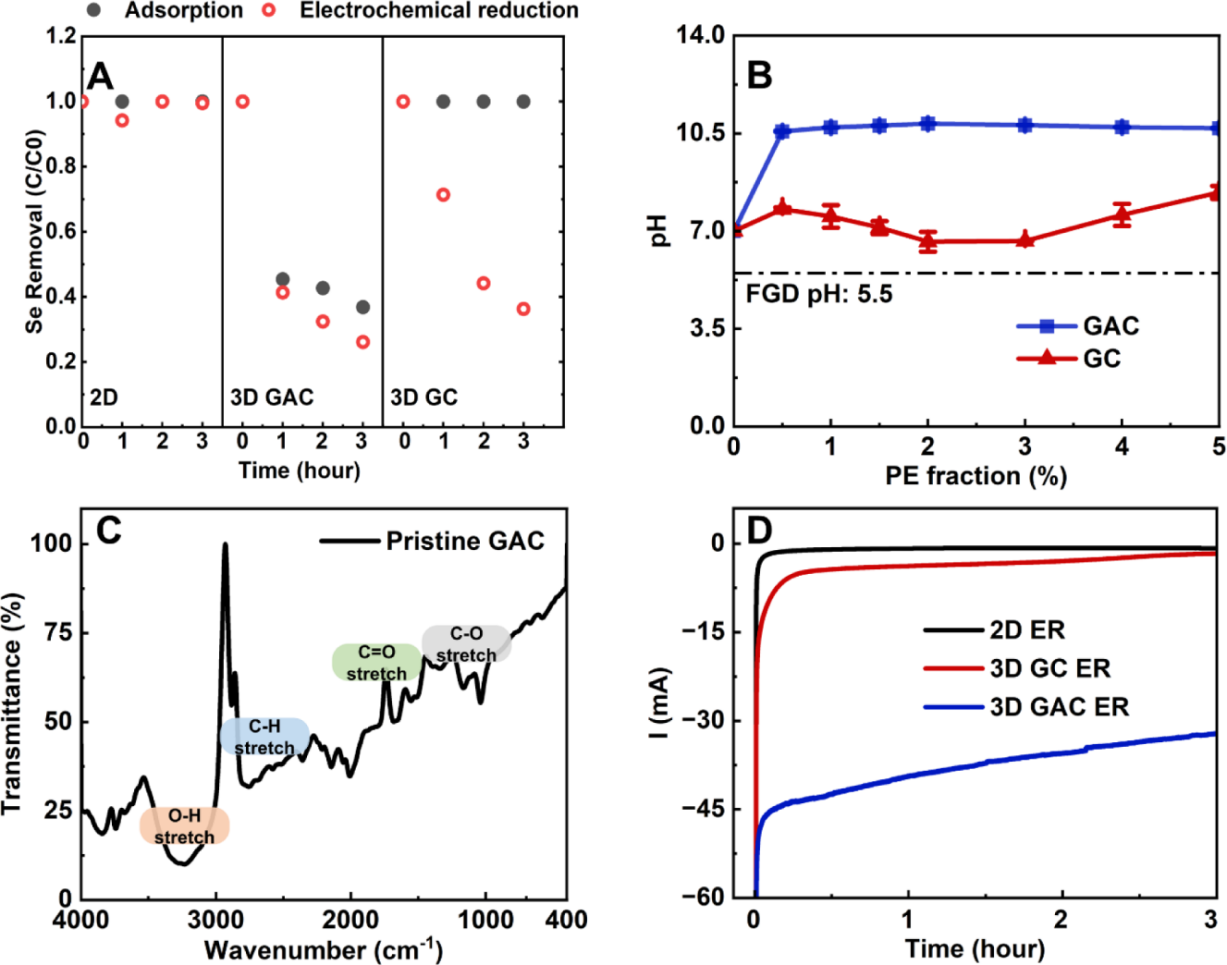
Performance comparison of 2D and 3DER in static operation mode. (A) Adsorption and direct electrochemical reduction performance of 2DER, 3DER-GAC, and 3DER-GC; (B) mass titration point of zero charge analysis for GAC and GC, with the PE fraction representing the weight percentage of PEs mixed with the aqueous solution. For example, 1% would be 1 g of GAC or GC in 100 mL of solution (SI, Table S2). The Y-axis indicates measured pH after mixing for 48 h; (C) FTIR of pristine GAC; and (D) current–time profile for 2DER, 3DER-GAC, and 3DER-GC. Experiments were triplicated, and graphs with error bars can be found in SI, Figure S7.

We then explored the direct electrochemical reduction of Se­(IV) at an *E*_cell_ of −1.9 V, assuming minimal electrosorption of Se­(IV) oxyanions due to the negatively biased cathode (*E*_we_ = −0.7 V). After 3 h, the 2DER exhibited negligible Se­(IV) removal despite a sufficient thermodynamic driving force for the Se­(IV)/Se­(−II) transformation to trigger subsequent Se(0) chemical precipitation (eq 5).^21,53^ This was likely due to its small RA/RV ratio and limited mass transfer in batch mode, which lacked mixing or stirring. While 3DER-GAC removed 73.8% of Se­(IV) in the same period, only 10.7% of this removal was attributed to electroreduction after accounting for its adsorption baseline. The large surface area of GAC resulted in high capacitance in its extensive double layer, leading to energy loss during charging and a high SEC of 0.61 kWh g^–1^ Se (Figure 1D). Moreover, the overlapping polarized double layers could block Se­(IV) from entering the GAC pores and reduce active electrode surface area, which results in low electrochemical removal of Se­(IV).^54–56^ On the other hand, the 3DER-GC removed slightly less Se­(IV) at 63.7% with an average removal rate of 1.62 mg L^–1^ h^–1^, primarily through electroreduction, as no adsorption was observed in the adsorption baseline. Notably, the 3DER-GC consumed 92% less energy (0.05 kWh g^–1^ Se) compared to the 3DER-GAC (0.63 kWh g^–1^ Se), owing to its adequate surface area, lower double-layer capacitance, and reduced non-Faradaic current (Figure 1D). Therefore, 3DER-GC provided the most energy-efficient removal of Se­(IV) via direct electrochemical reduction and was thus selected to further optimize mass transfer and reaction kinetics.

$$2HSe^{-}+HSeO_{3}^{-}+3H^{+}\to 3Se\left(0\right)+3H_{2}O$$ 5

### Dynamics of Electrochemical Impedance

We employed EIS and fit data into an equivalent circuit to identify the impedance components, including charge-transfer resistance (*R*_ct_), which reflects the ease of electron transfer at the electrode surface, and Warburg impedance (*Z*_w_), which represents the influence of mass transfer. The circuit model is chosen based on analysis of (i) our understanding and knowledge of the experimental system, (ii) the shape of the spectra at different frequency ranges, and (iii) similar reports in the literature (Supporting Information, Figure S10).^33,42^ Regarding the selection of the components and model of the circuit, we also verified that the error of the selected components and their numerical values was on the order of Chi-squared of 10^–3^. The Nyquist plot reveals that the initial intercept with the real axis (i.e., *x*-axis) at the highest frequency corresponds to equivalent series resistance (ESR) in the equivalent circuit, consisting of electrode resistance and electrolyte resistance (Figure 2A).^34,57^ The ESR is significantly lower in 3DER-GC (4.7 Ω) compared with 2DER (18.7 Ω, SI). This reduction is due to the GCs positioned between the two electrodes in 3DER, which provide much higher conductivity than the synthetic wastewater alone in 2DER. The reduced ESR facilitates a higher current flow at the same *E*_cell_, potentially accelerating the reaction kinetics. Following the ESR, the first semicircle on the Nyquist plot represents the parallel connection of the interparticle contact resistance (*R*_c_) and double-layer capacitance (represented as constant phase element CPE1) at the electrode–electrolyte interface. The diameter of the semicircle corresponds to *R*_c_, while its arc shape reflects the capacitive behavior of the double layer.^33,42^ Based on the fitted data in Table 1, decreasing *E*_cell_ from −1.9 to −2.3 V caused a negligible change in *R*_c_ (4.8 to 3.7 Ω), indicating *R*_c_ is primarily governed by the reactor’s physical property, i.e., the interparticle contact resistance of PEs. The capacitive component CPE1 accounts for the nonideal capacitance caused by the porous and irregular electrodes.^58,59^ Beyond this first semicircle describing the intrinsic property of the 3DER, the equivalent circuit includes Warburg impedance (*Z*_w_), representing the diffusion of ions in the electrolyte, along with a parallel combination of CPE2, which represents nonideal system-wide capacitance, and *R*_ct_ at the end of the circuit (Figure 2B). *R*_ct_ is sensitive to changes in *E*_cell_ and associated with electrochemical reactions such as Se­(IV) reduction and water splitting. It increased from 19.8 to 27.3 Ω as *E*_cell_ decreased from −1.9 to −2.3 V. This equivalent circuit analysis of σ and *R*_ct_ highlights the critical role of *E*_cell_ in governing the Se­(IV) removal performance.

**Figure 2:**
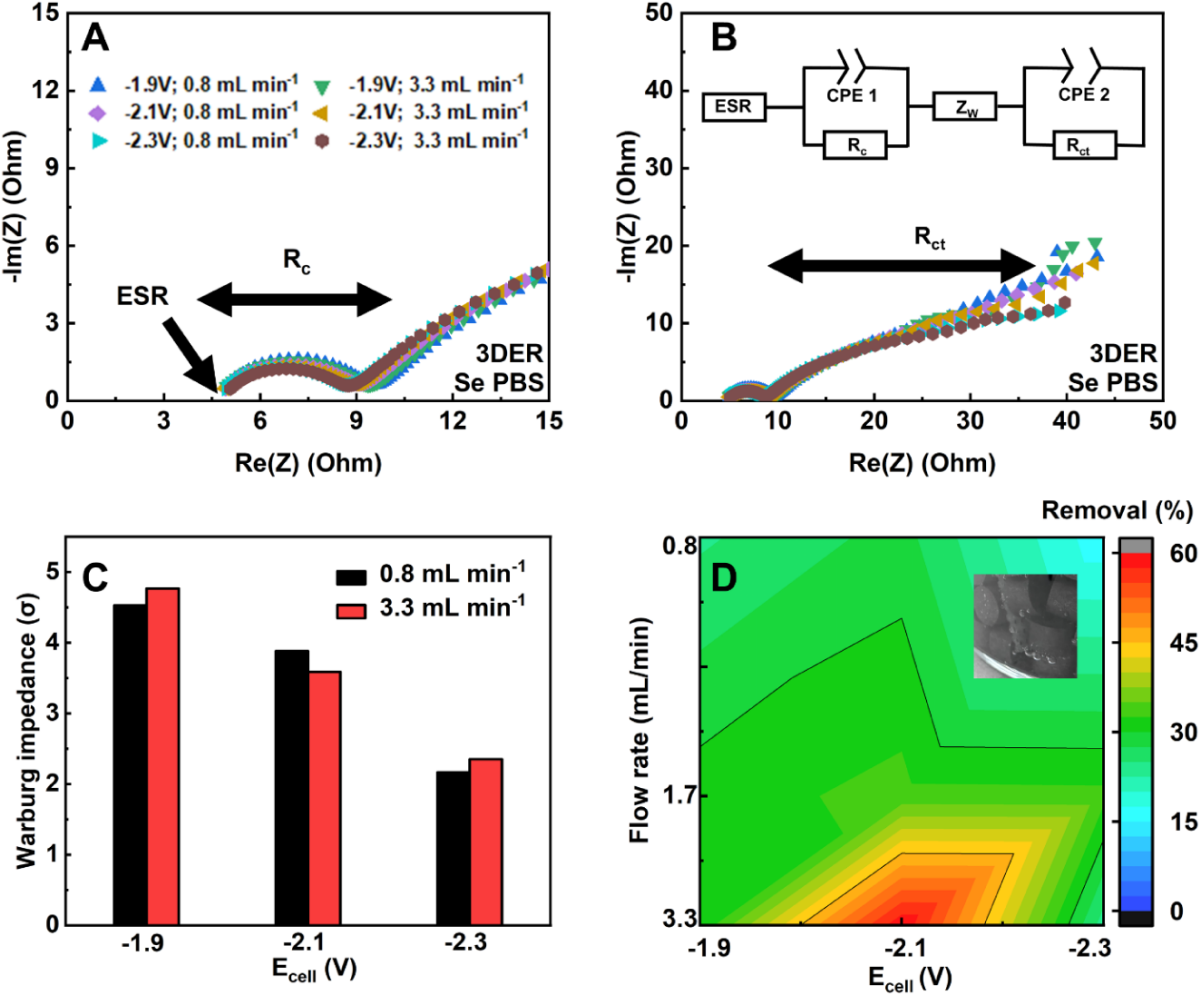
(A) Nyquist plot for zoomed-in EIS of 3DER under various operating conditions. The fitted equivalent circuit is provided, along with ESR and *R*_c_ labeled with arrows; (B) complete Nyquist plot for 3DER EIS with an equivalent circuit and *R*_ct_ labeled; (C) Warburg impedance σ from fitted EIS data against different recirculation rates and *E*_cell_; (D) Se­(IV) removal performance under various operating conditions, with an inset showing gas bubbles generated from water splitting sticking on the PE surface.

**Table 1:** Fitted Parameters from the Equivalent Circuit for 3DER Treating 0.1 mm Se­(IV) Synthetic Wastewater^a^

*E*_cell_ (V)	Recirc rate (mL min^–1^)	ESR (Ω)	*R*_c_ (Ω)	σ (Ω s^–1/2^)	*R*_ct_ (Ω)
–1.9 V	0.8	4.7	4.5	4.5	19.8
	3.3	4.7	4.4	4.8	18.0
–2.1 V	0.8	4.7	4.1	3.9	21.4
	3.3	4.7	3.9	3.6	23.3
–2.3 V	0.8	4.7	3.8	2.2	27.4
	3.3	4.8	3.7	2.4	25.2

a This table presents selected parameter values from fitted EIS measurements conducted under varying recirculation rates and *E*_cell_. The full set of parameters is available in SI, Table S3.

At the electrode–electrolyte interface, efficient diffusion is crucial for transporting target reactants such as Se­(IV) to the electrode surface. When diffusion is limited, the local concentration of redox-active species at the interface decreases, leading to concentration polarization, larger overpotentials, and reduced energy efficiency. To access diffusion conditions within the 3DER system, we extracted the Warburg coefficient (σ) by fitting EIS data to an equivalent circuit model incorporating Warburg impedance (*Z*_w_). Since the square value of σ (i.e., σ^2^) is inversely proportional to the diffusion coefficient, it serves as a useful indicator of effective mass transport.^34^ We collected EIS and performance data under different *E*_cell_ and recirculation rates to assess their impact on σ (Table 1). At a fixed recirculation rate of 0.8 mL min^–1^, σ continuously decreased from 4.5 to 2.1 Ω s^–1/2^, as *E*_cell_ shifted from −1.9 to −2.3 V. While the intrinsic diffusion coefficient of ions is not altered by *E*_cell_, this decrease in σ reflects enhanced effective mass transport at higher electric driving forces. Such apparent increases in diffusion coefficient may result from (1) thinning of the diffusion layer due to electroconvection, (2) additional migration contributions under stronger electric fields, and (3) convective effects induced by local density gradients, gas evolution, or electrohydrodynamic flows (Figure 2C).^60^ Despite these improvements in transport, Se­(IV) removal was lowest at −2.3 V (15.8% in 3 h) and highest at −2.1 V (28.5%, Figure 2D), showing that removal efficiency did not scale directly with enhanced effective diffusion and electric driving force. In contrast, increasing the recirculation rate from 0.8 to 3.3 mL min^–1^ did not produce remarkable changes in σ relative to the cell potential. Although slight variations in trend were observed across different potentials, these differences were likely to become negligible with additional experimental replicates. Experimentally, increasing the flow rate in general enhanced Se­(IV) removal, with performance boosted from 28.5% to 59.7% at an *E*_we_ of −2.1 V. These results demonstrate that the 3DER-GC is not strictly diffusion-limited, although mass transfer is significantly improved through forced convection, leading to enhanced Se­(IV) removal performance.

Beyond mass transfer, charge transfer resistance (*R*_ct_) at the electrode–electrolyte interface is another key factor governing reaction kinetics.^61^ In 3DER-GC, two processes could elevate *R*_ct_: (1) precipitation and accumulation of insulative Se(0) on the PEs and (2) gas bubble formation from water splitting. To investigate the effect of Se(0) accumulation, the *E*_cell_ and recirculation rate were held constant while the initial Se­(IV) concentration was increased from 0.1 to 10 mM. This change produced little variation in either ESR or *R*_ct_ across all recirculation rates, suggesting that the generated insulative Se(0) did not substantially precipitate or passivate the PE surface. Instead, the Se(0) particles remained suspended with the flow, preserving the interfacial conductivity and system performance. These results demonstrate that 3DER-GC can tolerate elevated Se­(IV) loadings, supporting its robustness in real-world applications. On the other hand, gas bubble accumulation from water splitting had a pronounced impact on charge transfer. At the highest *E*_cell_ of −2.3 V (*E*_we_ = −1.0 V, eq 6), bubbles adhered to the PEs, reducing the available electroactive surface area and thereby increasing *R*_ct_.^62^ This condition coincided with the lowest Se­(IV) removal (15.8% in 3 h), highest SEC (0.14 kWh g^–1^ Se), and lowest Faradaic efficiency (1.4%), at a recirculation rate of 0.8 mL min^–1^ and *R*_ct_ = 25.1 Ω. The Faradaic efficiency quantification method can be found in SI, eq S3–S5. By comparison, operating at an *E*_cell_ of −2.1 V and a higher recirculation rate (3.3 mL min^–1^) lowered *R*_ct_ to 23.2 Ω (Table 1), yielding the highest Faradaic efficiency (6.9%) and the lowest SEC (0.03 kWh g^–1^ Se). The increased recirculation rate likely destabilized gas bubbles from the PE surface, improved surface utilization, and Se­(IV) removal (Figure 2D). Taken together, EIS analysis and experimental results indicate that bubble accumulation from water splitting, rather than Se(0) precipitation, is the primary cause of elevated *R*_ct_ and performance decline at a high electric driving force. For optimal 3DER-GC operation, excessive *E*_cell_ should be avoided to balance the Se­(IV) removal efficiency with energy consumption. When high *E*_cell_ is unavoidable, increasing recirculation can mitigate bubble buildup and maintain efficient treatment performance.

6 $$2H_{2}O\left(l\right)\to 2H_{2}\left(g\right)+O_{2}\left(g\right),\left({E_{cell}}^{o}=-1.23V\right)$$

### Optimization of Reactor Configuration

Building on insights from EIS, we next optimized the 3DER configuration to enhance the Se­(IV) removal kinetics. In our 3DER, a plastic spacer separates the anodic and cathodic chambers to prevent short-circuiting. However, the spacer also introduces resistive losses (e.g., ohmic potential drop) and contributes to the overall system impedance. By repositioning this spacer, we altered the anode-to-cathode (A/C) chamber ratio, which in turn modified the potential distribution and the polarized surface area of the PEs in each chamber. Because Se­(IV) removal proceeds via cathodic reduction, we hypothesized that increasing the cathodic surface area would enhance its removal kinetics and efficiency.

We started with an A/C ratio of 2:1 (Figure 3A). Due to the low resistivity of the graphite cylinder PEs (50 μΩ·cm), the potential remained relatively uniform within each chamber, with most of the ohmic potential drop occurring across the spacer (Figure 3B).^63–65^ Under this configuration, the 3DER achieved linear Se­(IV) removal at 0.34 mg h^–1^ (*R* ^2^ = 0.9972) over 3 h, reaching 65.7% removal (Figure 3C). These linear removal kinetics indicate that the 3DER-GC system was not mass-transfer limited within the tested time frame, suggesting the extended operation could further lower the Se­(IV) concentration. The average SEC over 3 h is 0.02 kWh g^–1^ Se, and Faradaic efficiency increased from 6.5% in the first hour to 9.4% in the third hour.

**Figure 3:**
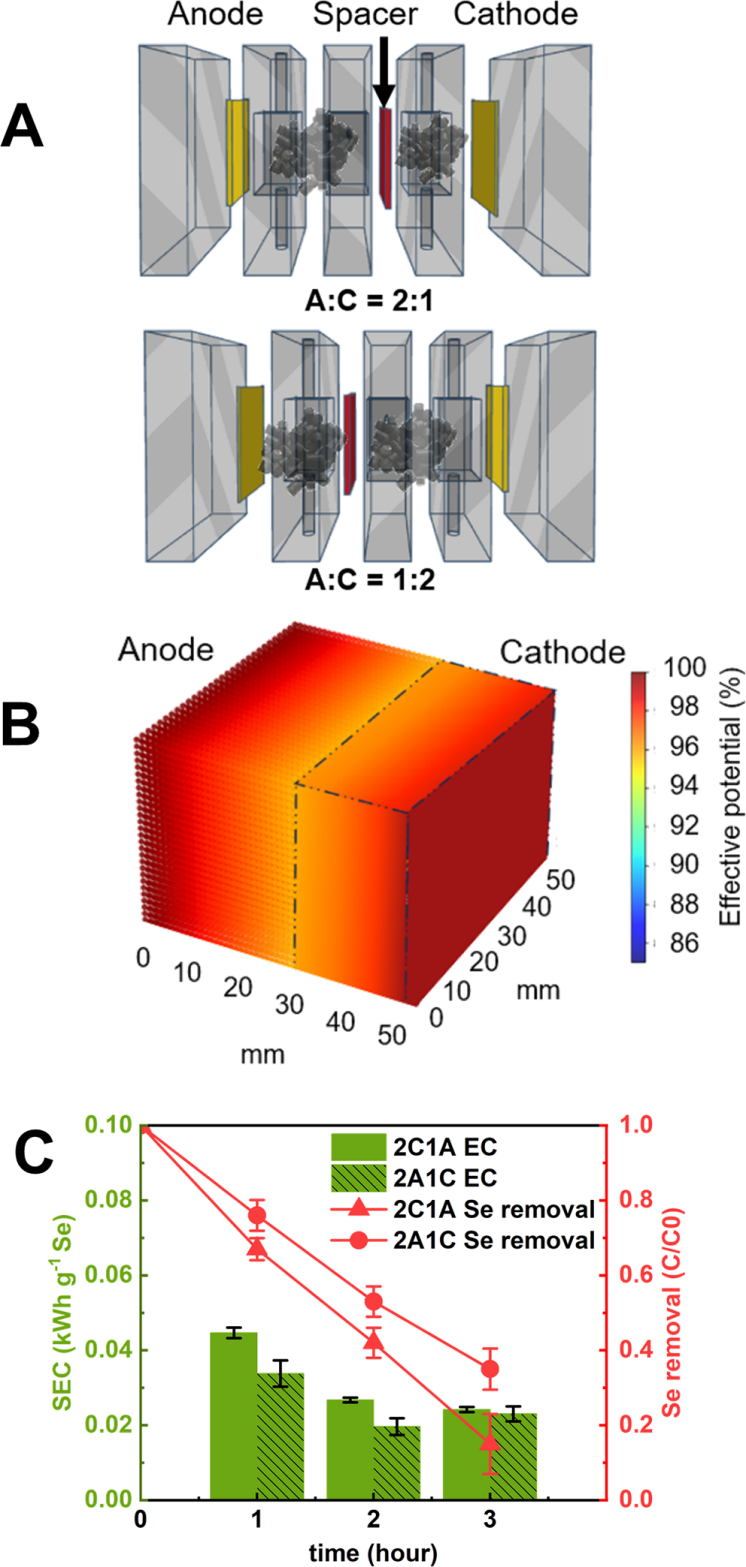
(A) 3DER setup with an anode-to-cathode chamber ratio (A:C) of 2:1 and 1:2; (B) illustration of effective potential distribution across the 3DER system based on simulation results, with an A:C = 2:1 configuration; and (C) energy consumption and Se­(IV) removal kinetics for 3DER configurations with A:C ratios of 2:1 and 1:2. Schematics for all 3DER configurations and corresponding simulations of effective potential distributions are provided in SI, Figure S4.

We then explored an extreme configuration (A:C = 1:10) by positioning the spacer adjacent to the graphite sheet anode, thereby maximizing cathode chamber volume and cathodic surface area while minimizing ohmic potential drop across the 3DER-GC chamber (SI, Figure S4). During a 12-h run, *E*_we_ dropped from −0.9 to −0.5 V, which is insufficient to sustain Se­(IV)/Se­(−II) reduction (*E*_we_ ≤ −0.6 V). At the same time, the *E*_ce_ increased from 1.2 to 1.6 V in 12 h, accelerating the oxygen evolution reaction and leading to severe surface oxidation and degradation of the graphite sheet anode.^66,67^ Consequently, Se­(IV) removal averaged only 10.8% after 12 h. Despite maximizing the cathodic chamber volume, this A:C = 1:10 configuration suffered from inadequate cathodic driving force and excessive anodic competing reactions.

To balance the Se­(IV) removal performance and electrode durability, we adopted the configuration with an A/C ratio of 1:2. This setup maintained *E*_we_ at −0.7 V, similar to the A:C = 2:1 configuration, while expanding the cathodic surface area. The Se­(IV) removal improved significantly, reaching 84.7% within 3 h (Figure 3C). The removal kinetics remained linear, with an average rate of 0.44 mg h^–1^, approximately 30% higher than the A:C = 2:1 configuration. Although the 3-h average SEC was 0.03 kWh g^–1^ Se (Figure 3C), the SEC value was only 0.02 kWh g^–1^ Se after the first hour as capacitive charging of PEs was completed. Given its enhanced Se­(IV) removal kinetics, desirable energy efficiency, and mitigated electrode degradation, the A/C = 1:2 setup was selected as our optimized 3DER configuration for subsequent single-pass experiments.

### Competing Ion Effects in Single-Pass FGD Treatment

While 3DER-GC demonstrated promising Se­(IV) removal efficiencies in a recirculating batch system, enhancing its treatment capacity and testing long-term stability are essential to manage FGD wastewater in coal-fired power plants. A single-pass, continuous-flow operation mode provides a more realistic assessment because fresh wastewater is continuously fed, simulating higher-throughput conditions. To maintain mass transfer comparable to the recirculation mode, an internal recirculation of 3.3 mL min^–1^ was applied during single-pass tests. We start operating 3DER-GC at a 1-h HRT, which is considerably shorter than the conventional biological treatment processes, e.g., 2–6 h of HRT for Veolia’s ABMet.^68,69^

When treating synthetic FGD wastewater, Se­(IV) removal in the single-pass effluent steadily improved over 12 h, increasing from 61.3% in the first hour to 68.1% by the 12th hour (Figure 4A). This trend supports our earlier conclusion that insulative Se(0) formation under the tested hydrodynamic conditions does not affect 3DER performance. Meanwhile, SEC decreased over time, from 0.08 kWh g^–1^ Se in the first hour to 0.02 kWh g^–1^ Se by the 12th hour (Figure 4B). This decrease is due to the completion of capacitive double-layer charging after the first hour, at which point the system stabilized at the target Se­(IV) reduction potential and maintained a steady current of 2.5 mA (Figure 4C).

**Figure 4:**
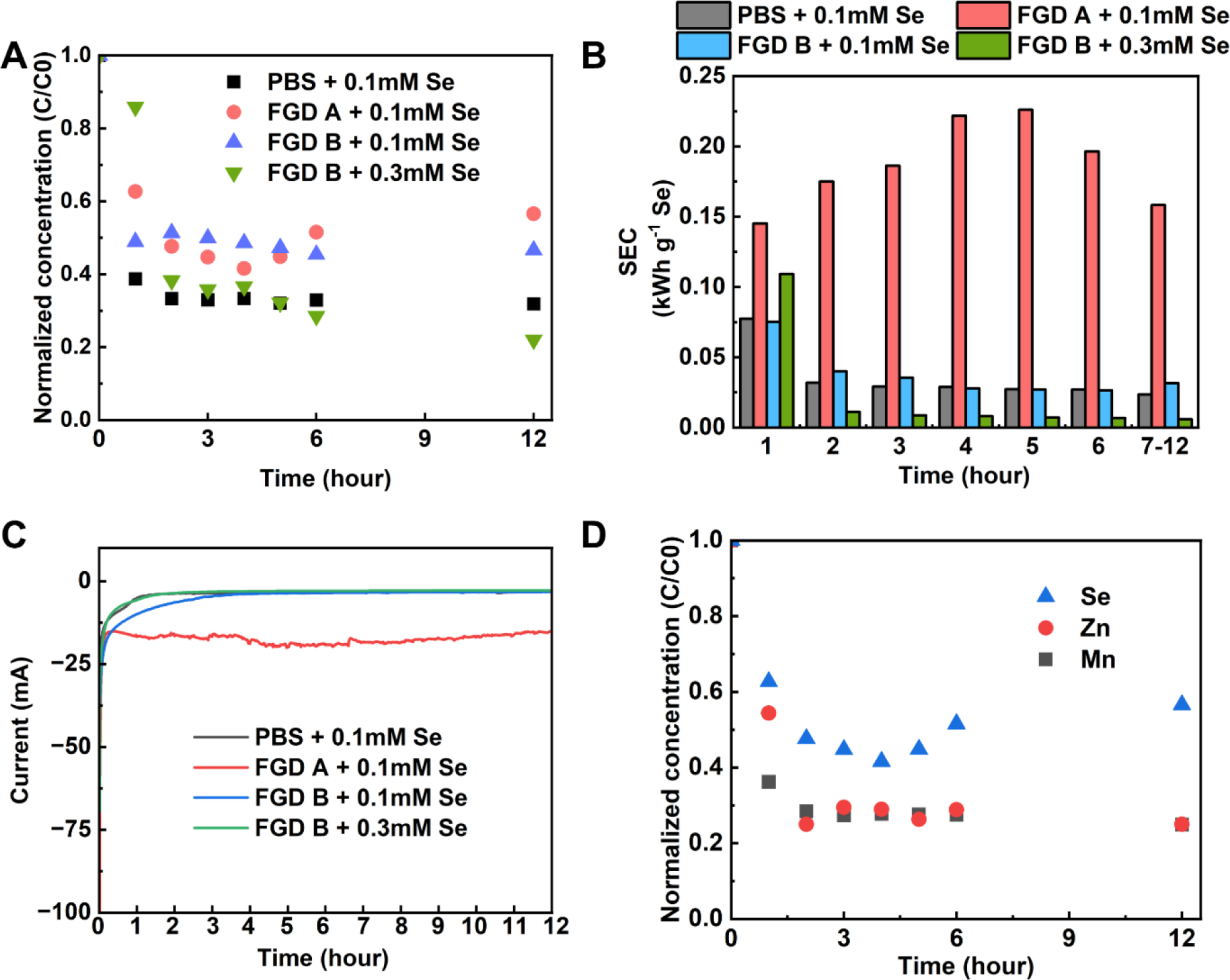
(A) Normalized concentration of Se­(IV) effluent of the single-pass 3DER system in a synthetic FGD water made of PBS and 0.1 mM Se­(IV), FGD-A spiked with 0.1 mM Se­(IV), and FGD-B spiked with either 0.1 or 0.3 mM Se­(IV); (B) hourly SCE treating the aforementioned four water matrices; (C) current–time (I–t) profiles of 3DER-GC in single-pass modes; and (D) normalized concentration of total soluble Se, Zn, and Mn from the single-pass 3DER system treating FGD-B spiked with 0.3 mM Se­(IV). Experiments were triplicated, and graphs with error bars can be found in SI, Figure S7.

Switching from synthetic wastewater to real FGD wastewater (i.e., FGD-A), Se­(IV) removal kinetics increased rapidly during the first 4 h, reaching an average efficiency of 60.4% and a maximum removal rate of 4.7 mg L^–1^ h^–1^. We also observed a similar increase in removal kinetics for several other cations present in real FGD wastewater, such as Mn and Zn (Figure 4D). This suggests early enhancement of Se­(IV) removal may result from electrochemical codeposition of multiple elements (e.g., MnSe or ZnSe formation). The influent Mn concentration in FGD-A was much higher (215.8 ppm or 3.9 mM) compared to Se­(IV) (7.8 ppm or 0.1 mM). After the single-pass treatment, effluent Mn dropped to 98.9 ppm or 1.8 mM, suggesting Mn removal via pathways other than MnSe formation (Table 2). A common product under these conditions is MnO_2_, an insulating precipitate that can reduce the electroactive surface area and alter the local pH/potential.^65,70^ We did not observe a continuous drop in the working current during 12 h, although Mn signals were detected on PE surfaces by SEM-EDS (SI, Figure S5).

**Table 2:** Average Conductivity and Concentration Profiles of Total Cationic and Anionic Species in Synthetic and Real FGD Wastewater^a^

	Synthetic (8.7 ± 0.1 mS cm^–1^) (ppm)	FGD-A (14.3 ± 0.2 mS cm^–1^) (ppm)	FGD-B (10.8 ± 0.2 mS cm^–1^) (ppm)
Mn	0	215.8 ± 21.1	3.3 ± 0.1
Si	0	28.9 ± 4.1	50 ± 5.9
K	63.9 ± 5.8	240.2 ± 28.1	88.2 ± 12.9
Ca	0	10176.7 ± 298.3	774.3 ± 12.7
Mg	0	4403.4 ± 510.3	1338.4 ± 217.8
Cl	0	28583.2 ± 1740.3	2968.3 ± 361.8

a A comprehensive sample composition analysis can be found in SI, Table S1.

High concentrations of background cations in FGD-A can also compete for cathodic surface sites, shielding Se­(IV) from electrode contact and decreasing selectivity.^70,71^ In addition to cations, anions such as chlorine (Cl^–^) may further hinder Se­(IV) reduction.^17,29,72^ The chlorine evolution reaction generates reactive chlorine species that can reoxidize Se­(−II) back to Se­(IV).^73,74^ This side reaction likely contributes to a decreased overall removal efficiency, particularly for the Se­(IV)/Se­(−II) reduction pathway.^75^ Note that high chloride concentrations also challenge biological treatment processes, in which multistage bioreactors to lower redox potential before Se­(IV) reduction can occur are often required.^76^ Competing ion tests in 3DER with synthetic wastewater spiked as little as 2900 ppm (250 mM). Cl^–^ confirmed complete inhibition of Se­(IV)/Se­(−II) reduction (SI, Figure S6), as the Cl^–^ concentration is 2500× that of the Se concentration (0.1 mM), and the chlorine evolution parasitic reaction completely terminated the Se reduction pathway. In contrast, although FGD-A contained 28583.2 ppm of Cl^–^, it sustained Se reduction over 12 h of continuous operation. The high salinity provided high conductivity, which resulted in a strong driving force for the Se­(IV) reduction reaction (Figure 4c). The FGD-A diverse ion composition could also suppress the selectivity of anode PEs toward chloride ions by introducing other interfacial reactions, such as the deposition of Mn and Si onto the electrode surface. Manganese oxide (E_MnOx/Mn2+_° = 0.3 V vs Ag/AgCl) and silicon oxides have been reported to form selective permeable layers on the electrodes, which block Cl^–^ from contacting the electrode surface, thus enhancing OER selectivity over CLER.^77–79^ The high concentrations of Mn and Si in the FGD wastewater could allow partial Se­(IV) reduction compared to synthetic FGD wastewater with only chloride added (Table 2). Unfortunately, this complex background ion composition of FGD-A also resulted in decreased Se­(IV) selectivity and a six times higher energy consumption (0.19 kWh g^–1^ Se) compared to treating synthetic FGD wastewater (Figure 4B).

To further probe matrix effects, we tested FGD-B with different background ion concentrations, where most of the ions have a lowered concentration compared to FGD-A. Here, Se­(IV) removal efficiency increased slightly to 51.7% or 4.2 mg L^–1^ h^–1^, compared to 49.8% when treating FGD-A (Figure 4A). Importantly, SEC dropped substantially from 0.07 to 0.03 kWh g^–1^ Se, highlighting that the reduced ion competition outweighed the effect of lower solution conductivity. We further examined the 3DER-GC performance under an elevated Se­(IV) loading (0.3 mM) in FGD-B. At this concentration, the 3DER performance improved significantly, with hourly average Se­(IV) removal at 62.3% (or 14.6 mg L^–1^ h^–1^) and kinetics increased over time (Figure 4A). By the 12th hour, 3DER reached its peak hourly removal of 74.4% (or 17.5 mg L^–1^ h^–1^), suggesting that the system was not mass-transfer limited and that continuous Se(0) formation did not impair removal performance. SEC averaged 0.02 kWh g^–1^ Se, the lowest among all wastewater matrices tested (Figure 4B). Collectively, these results demonstrate the robustness of 3DER-GC under complex water matrices and elevated Se loadings. This system maintained effective Se­(IV) removal without pH adjustment or chemical additives, achieving rapid Se­(IV) reduction across multiple real FGD wastewaters. Although the effluent Se­(IV) concentration may still exceed the regulatory discharge limit, we want to emphasize that the experiment is conducted with minimally pretreated FGD wastewater.^8^ Integrating 3DER in a treatment train can effectively lower the Se­(IV) loading for subsequent treatment processes and can serve as a safety measure to lower Se concentrations even when downstream treatment processes face operational challenges in harsh or chemically challenging water matrices.^80,81^ These findings highlight the potential of 3DER-GC as a practical, chemical-free, and efficient technology for treating FGD and other complex industrial wastewaters.

## Conclusions

This study demonstrates that the optimized 3DER, incorporating graphite cylinder PEs, controlled recirculation, and tuned cell potentials, enabled efficient Se­(IV) removal across both synthetic and real FGD wastewater matrices. In synthetic wastewater containing 0.1 mM Se­(IV), the 3DER-GC exhibited stable removal performance over 12 h in single-pass mode, achieving a peak hourly removal of 68.1% and a minimum SEC of 0.02 kWh g^–1^ Se as the system reached steady-state operation. In real FGD wastewater, the 3DER-GC maintained average hourly Se­(IV) removals of 51.7% in FGD-B and 49.8% in FGD-A, despite substantially higher background ion concentrations. Although the removal efficiency was not markedly impacted, energy use remained matrix-dependent, with more complex water matrices exhibiting higher SEC. While elevated chloride strongly inhibited Se­(IV) reduction in simplified synthetic FGD matrices, the presence of additional constituents in real FGD wastewater, such as Mn and Si, can suppress chloride evolution and enable partial Se­(IV) removal compared to synthetic wastewater containing only chloride. Nonetheless, Cl^–^ remained a critical limiting factor, underscoring the need for electrode materials with higher chloride evolution potentials or for pretreatment strategies capable of reducing elevated chloride levels. Overall, our results demonstrate the robustness and scalability of the 3DER system. With continued advancements in electrode and spacer design to improve selectivity, suppress parasitic reactions, and lower energy intensity, the 3DER shows strong promise as a practical treatment technology for selenium-rich FGD wastewater and other high-salinity industrial effluents.

## Supplementary Material



## Notes

Reactor configuration; validation of Se­(IV) 6-e reduction pathway in 3DER; validation of cleaning protocol; FGD wastewater full spectrum analysis; E_we_ vs time recorded data; mass titration method for point of zero charge analysis; electrochemical impedance analysis for 2DER and 3DER-GC; schematic and effective potential simulations for 3DER-GC under different chamber ratios; SEM/EDS mapping of PE surface and precipitates; competing ion experiment in synthetic wastewater; triplicated experiment data with error bars; Faradaic efficiency quantification; equivalent circuit selection for 3DER EIS (PDF)

## References

[1] NIH. Selenium Fact Sheet for Consumers; NIH 2021.

[2] Murray C. B., Norris D. J., Bawendi M. G. (1993). Synthesis and Characterization of
Nearly Monodisperse CdE (E = S, Se, Te) Semiconductor Nanocrystallites. J. Am. Chem. Soc..

[3] Aro A., Alfthan G., Varo P. (1995). Effects of
Supplementation of Fertilizers
on Human Selenium Status in Finland. Analyst.

[4] Lemly A. D. (2004). Aquatic
Selenium Pollution Is a Global Environmental Safety Issue. Ecotoxicol. Environ. Saf..

[5] Etteieb S., Magdouli S., Zolfaghari M., Brar S. K. (2020). Monitoring and Analysis
of Selenium as an Emerging Contaminant in Mining Industry: A Critical
Review. Sci. Total Environ..

[6] Cordoba P., Staicu L. C. (2018). Flue Gas Desulfurization Effluents: An Unexploited
Selenium Resource. Fuel.

[7] Gingerich D. B., Grol E., Mauter M. S. (2018). Fundamental
Challenges and Engineering
Opportunities in Flue Gas Desulfurization Wastewater Treatment at
Coal Fired Power Plants. Environ. Sci. Water
Res. Technol..

[8] U.S. Environmental Protection Agency. Steam electric power generating point source category: Final detailed study report; U.S. Environmental Protection Agency, 2017.

[9] Henry B. L., Wesner J. S., Kerby J. L. (2020). Cross-Ecosystem
Effects of Agricultural
Tile Drainage, Surface Runoff, and Selenium in the Prairie Pothole
Region. Wetlands.

[10] Gene S. M., Hoekstra P. F., Hannam C., White M., Truman C., Hanson M. L., Prosser R. S. (2019). The Role
of Vegetated Buffers in
Agriculture and Their Regulation across Canada and the United States. J. Environ. Manage..

[11] U.S. Environmental Protection Agency. 33 U.S.C. § 1344; U.S. Environmental Protection Agency, 1972.

[12] Ečimović S., Velki M., Vuković R., Štolfa Čamagajevac I., Petek A., Bošnjaković R., Grgić M., Engelmann P., Bodó K., Filipović-Marijić V., Ivanković D., Erk M., Mijošek T., Lončarić Z. (2018). Acute Toxicity of Selenate and Selenite
and Their Impacts on Oxidative Status, Efflux Pump Activity, Cellular
and Genetic Parameters in Earthworm Eisenia Andrei. Chemosphere.

[13] Li J., Loi G., Otero-Gonzalez L., Laing G. D., Ferrer I., Lens P. N. L. (2020). Selenate and
Selenite Uptake, Accumulation and Toxicity
in Lemna Minuta. Water Sci. Technol..

[14] Zhang N., Lin L. S., Gang D. (2008). Adsorptive Selenite Removal from
Water Using Iron-Coated GAC Adsorbents. Water
Res..

[15] Sun H. J., Rathinasabapathi B., Wu B., Luo J., Pu L. P., Ma L. Q. (2014). Arsenic and Selenium Toxicity and
Their Interactive Effects in Humans. Environ.
Int..

[16] Liang L., Yang W., Guan X., Li J., Xu Z., Wu J., Huang Y., Zhang X. (2013). Kinetics and Mechanisms of PH-Dependent
Selenite Removal by Zero Valent Iron. Water
Res..

[17] Zou S., Mauter M. S. (2021). Competing Ion Behavior in Direct Electrochemical Selenite
Reduction. ACS ES T Eng..

[18] Tang C., Huang Y. H., Zeng H., Zhang Z. (2014). Reductive Removal of
Selenate by Zero-Valent Iron: The Roles of Aqueous Fe2+ and Corrosion
Products, and Selenate Removal Mechanisms. Water
Res..

[19] Zou S., Mauter M. S. (2021). Direct
Electrochemical Pathways for Selenium Reduction
in Aqueous Solutions. ACS Sustain. Chem. Eng..

[20] Guarneros-Aguilar C., Calzadilla O., Barón-Miranda J. A., Fernandez-Munoz J. L., Caballero-Briones F. (2019). Phase Control in Selenium Electrodeposition with Bath
Temperature and Deposition Potential. Mater.
Res. Express.

[21] Yang Z., Zhao J., Sullivan E. G., Zou S. (2023). Cost-Effective
Cathode
Materials To Electrochemically Tackle Aquatic Selenite Pollution. ACS ES T Eng..

[22] Norra G. F., Radjenovic J. (2021). Removal of Persistent Organic Contaminants
from Wastewater
Using a Hybrid Electrochemical-Granular Activated Carbon (GAC) System. J. Hazard. Mater..

[23] Ju Y., Liu C., Ganiyu S. O., Zhao Y., Gamal El-Din M. (2023). Electrochemical
Degradation of Dissolved Organic Matters in Oil Sands Process Water
Using Continuous-Flow Packed Bed Electrode Reactor. Sep. Purif. Technol..

[24] Samarghandi M. R., Ansari A., Dargahi A., Shabanloo A., Nematollahi D., Khazaei M., Nasab H. Z., Vaziri Y. (2021). Enhanced Electrocatalytic
Degradation of Bisphenol A by Graphite/β-PbO2 Anode in a Three-Dimensional
Electrochemical Reactor. J. Environ. Chem. Eng..

[25] Tezcan
Un U., Aytac E. (2013). Electrocoagulation in a Packed Bed Reactor-Complete
Treatment of Color and Cod from Real Textile Wastewater. J. Environ. Manage..

[26] Ren X., Tang P., Xu L., Zhang Q., Song K., Pan Z. (2022). Review on Treating Refractory, Organics-Laden Wastewater Using Three-Dimensional
Electrochemical Reactor. Desalin. Water Treat..

[27] Ye W., Zhang W., Hu X., Yang S., Liang W. (2020). Efficient
Electrochemical-Catalytic Reduction of Nitrate Using Co/AC0.9-AB0.1
Particle Electrode. Sci. Total Environ..

[28] Zhang Y., Zhang D., Zhou L., Zhao Y., Chen J., Chen Z., Wang F. (2018). Polypyrrole/Reduced
Graphene Oxide
Aerogel Particle Electrodes for High-Efficiency Electro-Catalytic
Synergistic Removal of Cr­(VI) and Bisphenol A. Chem. Eng. J..

[29] Cho S., Kim C., Hwang I. (2020). Electrochemical Degradation of Ibuprofen
Using an Activated-Carbon-Based
Continuous-Flow Three-Dimensional Electrode Reactor (3DER). Chemosphere.

[30] Goulet M., Skyllas-Kazacos M., Kjeang E. (2016). The Importance of Wetting in Carbon
Paper Electrodes for Vanadium Redox Reactions. Carbon.

[31] Coleman L., Bragg L. J., Finkelman R. B. (1993). Distribution
and Mode of Occurrence
of Selenium in US Coals. Environ. Geochem. Health.

[32] Gingerich D. B., Grol E., Mauter M. S. (2018). Fundamental
Challenges and Engineering
Opportunities in Flue Gas Desulfurization Wastewater Treatment at
Coal Fired Power Plants. Environ. Sci.: water
Res. Technol..

[33] Bredar A. R. C., Chown A. L., Burton A. R., Farnum B. H. (2020). Electrochemical
Impedance Spectroscopy of Metal Oxide Electrodes for Energy Applications. ACS Appl. Energy Mater..

[34] Xie A., Martínez-Vargas D. R., Yang Z., Zou S. (2024). Efficient
Selenate Removal from Impaired Waters with TiO2-Assisted Electrocatalysis. Water Res..

[35] American Public Health Association American Water Works. Standard Methods For The Examination Of Water And Wastewater.; American Public Health Association American Water Works, 2023

[36] Menéndez J. A., Illán-Gómez M. J., YLeón C. A. L., Radovic L. R. (1995). On the Difference between the Isoelectric Point and
the Point of Zero Charge of Carbons. Carbon.
Carbon..

[37] Reymond J. P., Kolenda F. (1999). Estimation of the Point of Zero Charge of Simple and
Mixed Oxides by Mass Titration. Powder Technol..

[38] Shin S., Jang J., Yoon S. H., Mochida I. (1997). A Study on the Effect
of Heat Treatment on Functional Groups of Pitch Based Activated Carbon
Fiber Using FTIR. Carbon N. Y.

[39] Parbhoo, B. ; O’Hare, L.-A. ; Leadley, S. R. Chapter 14 - Fundamental Aspects of Adhesion Technology in Silicones. In Adhesion Science and Engineering; Elsevier, 2002.

[40] Wasewar K. L., Prasad B., Gulipalli S. (2009). Removal of Selenium by Adsorption
onto Granular Activated Carbon (GAC) and Powdered Activated Carbon
(PAC). Clean - Soil, Air, Water.

[41] Jung M. W., Ahn K. H., Lee Y., Kim K. P., Rhee J. S., Park J. T., Paeng K. J. (2001). Adsorption Characteristics of Phenol
and Chlorophenols on Granular Activated Carbons (GAC). Microchem. J..

[42] Zárate-Guzmán A. I., Manríquez-Rocha J., Antaño-López R., Rodríguez-Valadez F. J., Godínez L. A. (2018). Study of
the Electrical Properties of a Packed Carbon Bed for Its Potential
Application as a 3D-Cathode in Electrochemical Processes. J. Electrochem. Soc..

[43] Hubicki Z., Barczak M. (2005). Adsorption of Phenolic Compounds by Activated Carbon
— a Critical Review. Chemosphere.

[44] Mene J. A., Xia B., Phillips J., Radovic L. R. (1997). On the Modification and Characterization
of Chemical Surface Properties of Activated Carbon: Microcalorimetric,
Electrochemical, and Thermal Desorption Probes. Langmuir.

[45] Online V.
A., Liu B., Li H., Chen R., Wang S., Li L. (2018). The Synergistic
Effect of Oxygen-Containing Functional Groups on CO_2_ Adsorption
by the glucose–potassium citrate-derived activated carbon. RSC Adv..

[46] Liang C., Lee P. (2012). Granular Activated
Carbon/Pyrite Composites for Environmental Application:
Synthesis and Characterization. J. Hazard. Mater..

[47] Susanto S., Nurtono T., Widiyastuti W., Yeh M., Setyawan H. (2024). Controlling
N-Doping Nature at Carbon Aerogels from Biomass for Enhanced Oxygen
Reduction in Seawater Batteries. ACS Omega.

[48] Sun Y., Jia J., Liu Z., Liu Z., Huo L., Zhao L., Zhao Y., Yao Z. (2023). Heteroatom
- Doped Biochar Devised
from Cellulose for CO 2 Adsorption: A New Vision on Competitive Behavior
and Interactions of N and S. Biochar.

[49] Xing W., Ngo H. H., Kim S. H., Guo W. S., Hagare P. (2008). Adsorption
and Bioadsorption of Granular Activated Carbon (GAC) for Dissolved
Organic Carbon (DOC) Removal in Wastewater. Bioresour. Technol..

[50] Belkouteb N., Franke V., McCleaf P., Köhler S., Ahrens L. (2020). Removal of Per- and Polyfluoroalkyl
Substances (PFASs)
in a Full-Scale Drinking Water Treatment Plant: Long-Term Performance
of Granular Activated Carbon (GAC) and Influence of Flow-Rate. Water Res..

[51] Hijnen W. A. M., Suylen G. M. H., Bahlman J. A., Brouwer-Hanzens A., Medema G. J. (2010). GAC Adsorption Filters as Barriers
for Viruses, Bacteria
and Protozoan (Oo)­Cysts in Water Treatment. Water Res..

[52] Velten S., Boller M., Köster O., Helbing J., Weilenmann H. U., Hammes F. (2011). Development of Biomass
in a Drinking Water Granular
Active Carbon (GAC) Filter. Water Res..

[53] Kim Y. J., Yuan K., Ellis B. R., Becker U. (2017). Redox Reactions of
Selenium as Catalyzed by Magnetite: Lessons Learned from Using Electrochemistry
and Spectroscopic Methods. Geochim. Cosmochim.
Acta.

[54] Das S., Chakraborty S. K., Mitra S. K. (2012). Redefining Electrical Double Layer
Thickness in Narrow Confinements: Effect of Solvent Polarization. Phys. Rev. E.

[55] Yang K., Ying T., Yiacoumi S., Tsouris C. (2008). Electrosorption of
Ions from Aqueous Solutions by Carbon Aerogel: An Electrical Double-Layer
Model. Langmuir.

[56] Shi H. (1996). Activated
Carbons and Double Layer Capacitance. Electrochim.
Acta.

[57] Lazanas A. C., Prodromidis M. I. (2023). Electrochemical Impedance Spectroscopy–A Tutorial. ACS Meas. Sci. Au.

[58] Gateman S. M., Gharbi O., Gomes de
Melo H., Ngo K., Turmine M., Vivier V. (2022). On the Use of a Constant
Phase Element (CPE) in Electrochemistry. Curr.
Opin. Electrochem..

[59] Jorcin J. B., Orazem M. E., Pébère N., Tribollet B. (2006). CPE Analysis
by Local Electrochemical Impedance Spectroscopy. Electrochim. Acta.

[60] Baur, J. E. Handbook of Electrochemistry; Elsevier, 2007.

[61] Lao M., Li P., Jiang Y., Pan H., Dou S., Sun W. (2022). From Fundamentals
and Theories to Heterostructured Electrocatalyst Design: An in-Depth
Understanding of Alkaline Hydrogen Evolution Reaction. Nano Energy.

[62] Primdahl S., Mogensen M. (1999). Gas Diffusion Impedance
in Characterization of Solid
Oxide Fuel Cell Anodes. J. Electrochem. Soc..

[63] Sengupta R., Bhattacharya M., Bandyopadhyay S., Bhowmick A. K. (2011). A Review on the
Mechanical and Electrical Properties of Graphite and Modified Graphite
Reinforced Polymer Composites. Prog. Polym.
Sci..

[64] Zhu X., Jassby D. (2019). Electroactive Membranes
for Water Treatment: Enhanced
Treatment Functionalities, Energy Considerations, and Future Challenges. Acc. Chem. Res..

[65] Ma S., Yang F., Chen X., Khor C. M., Jung B., Iddya A., Sant G., Jassby D. (2021). Removal of As­(III)
by Electrically Conducting Ultrafiltration Membranes. Water Res..

[66] Choo H. S., Kinumoto T., Nose M., Miyazaki K., Abe T., Ogumi Z. (2008). Electrochemical Oxidation
of Highly Oriented Pyrolytic Graphite during
Potential Cycling in Sulfuric Acid Solution. J. Power Sources.

[67] Qiao M. X., Zhang Y., Zhai L. F., Sun M. (2018). Corrosion of Graphite
Electrode in Electrochemical Advanced Oxidation Processes: Degradation
Protocol and Environmental Implication. Chem.
Eng. J..

[68] Sinharoy A., Lens P. N. L. (2020). Biological Removal of Selenate and Selenite from Wastewater:
Options for Selenium Recovery as Nanoparticles. Curr. Pollut. Rep..

[69] Ali I., Shrivastava V. (2021). Recent Advances in Technologies for Removal and Recovery
of Selenium from (Waste)­Water: A Systematic Review. J. Environ. Manage..

[70] Fan J., Arrazolo L. K., Du J., Xu H., Fang S., Liu Y., Wu Z., Kim J. H., Wu X. (2024). Effects of Ionic Interferents
on Electrocatalytic Nitrate Reduction: Mechanistic Insight. Environ. Sci. Technol..

[71] Zheng C., Ji D., Yao Q., Bai Z., Zhu Y., Nie C., Liu D., Wang N., Yang J., Dou S. (2023). Electrostatic Shielding
Boosts Electrochemical Performance of Alloy-Type Anode Materials of
Sodium-Ion Batteries. Angew. Chem. Int. Ed..

[72] Holmes A. B., Ngan A., Ye J., Gu F. (2022). Selective Photocatalytic
Reduction of Selenate over TiO2 in the Presence of Nitrate and Sulfate
in Mine-Impacted Water. Chemosphere.

[73] Karlsson R. K. B., Cornell A. (2016). Selectivity between
Oxygen and Chlorine Evolution in
the Chlor-Alkali and Chlorate Processes. Chem.
Rev..

[74] Liu S., Salhi E., Huang W., Diao K., von Gunten U. (2019). Kinetic and
Mechanistic Aspects of Selenite Oxidation by Chlorine, Bromine, Monochloramine,
Ozone, Permanganate, and Hydrogen Peroxide. Water Res..

[75] Hao S., Feng Y., Wang D., Cho J., Qiu C., Wi T. U., Xu Z., Yu Z., Sellers C., Zou S. (2025). Electrochemical Removal
of Se­(IV) from Wastewater Using
RuO2-Based Catalysts. Nano Lett..

[76] Sonstegard J. (2009). ABMet: Setting
the Standard for Selenium Removal. Proc. Water
Environ. Fed..

[77] Vos J. G., Wezendonk T. A., Jeremiasse A. W., Koper M. T. M. (2018). MnOx/IrOx as
Selective Oxygen Evolution Electrocatalyst in Acidic Chloride Solution. J. Am. Chem. Soc..

[78] Najafpour M. M., Renger G., Hołyńska M., Moghaddam A. N., Aro E. M., Carpentier R., Nishihara H., Eaton-Rye J. J., Shen J. R., Allakhverdiev S. I. (2016). Manganese
Compounds as Water-Oxidizing Catalysts: From the Natural Water-Oxidizing
Complex to Nanosized Manganese Oxide Structures. Chem. Rev..

[79] Bhardwaj A. A., Vos J. G., Beatty M. E. S., Baxter A. F., Koper M. T. M., Yip N. Y., Esposito D. V. (2021). Ultrathin Silicon
Oxide Overlayers
Enable Selective Oxygen Evolution from Acidic and Unbuffered PH-Neutral
Seawater. ACS Catal..

[80] Igunnu E. T., Chen G. Z. (2014). Produced Water Treatment
Technologies. Int. J. Low-Carbon Technol..

[81] Garcia-Rodriguez O., Mousset E., Olvera-Vargas H., Lefebvre O. (2022). Electrochemical Treatment
of Highly Concentrated Wastewater: A Review of Experimental and Modeling
Approaches from Lab- to Full-Scale. Crit. Rev.
Environ. Sci. Technol..

