# Supplementary material for: Three-Dimensional Packed-Bed Electrochemical Reactor Design for Selective Selenite Reduction in Water
Source: ACS ES T Eng. 2025 Dec 29;6(1):404–15. doi: 10.1021/acsestengg.5c00861 (PMC12797224; doi:10.1021/acsestengg.5c00861)
Supplement: Supplementary file 1 [file ee5c00861_si_001.pdf]

Supporting Information for

Three-dimensional packed bed electrochemical reactor design for selective selenite reduction in water

Zilan Yang,[a] D. Ricardo Martinez-Vargas,[a] Ao Xie,[a] Shengcun Ma,[b] Shiqiang Zou[a,*]

[a] Department of Civil and Environmental Engineering, Michigan State University, East Lansing, Michigan 48824, USA
[b] Energy Storage and Distributed Resources Division, Lawrence Berkeley National Laboratory, Berkeley, California 94720, United States

[*]Corresponding author.
Shiqiang Zou. E-mail: zoushiqi@msu.edu

**Total pages:**	**15**
**Total figures:**	**10**
**Total tables:**	**3**
**Total equations:**	**5**

1. Reactor configuration

2D or 3DER is built with 5 acrylic plates with dimensions of 10 x 10 cm, the 2 end plates have 1 cm thickness, while the center 3 plates have a thickness of 1.5 cm. Rubber gaskets are placed between the plates to prevent leakage and secure components such as graphite sheet electrodes and the spacer. A perforated plastic spacer is secured between the plates to separate the PEs and prevent short circuit, while allowing continuous flow of solution.

The overall chamber size is 5 x 5 x 5 cm, with thickness of 3 plates (1.5x3=4.5cm) and 5 middle rubber gaskets (0.1x5=0.5cm). The two gaskets at the end are not carved and do not result in additional reactor volume. The measured chamber volume is 120 mL, slightly less than the theoretical 125 mL, due to the spacer and possible imperfection in gasket carving.

For the 3DER, the chamber is filled with GAC and graphite PEs. The PEs are tightly in contact with the graphite sheet terminal electrodes and can conduct necessary potential for Se(IV) reduction. 3DER chamber volume is reduced to 50 mL due to the PEs.

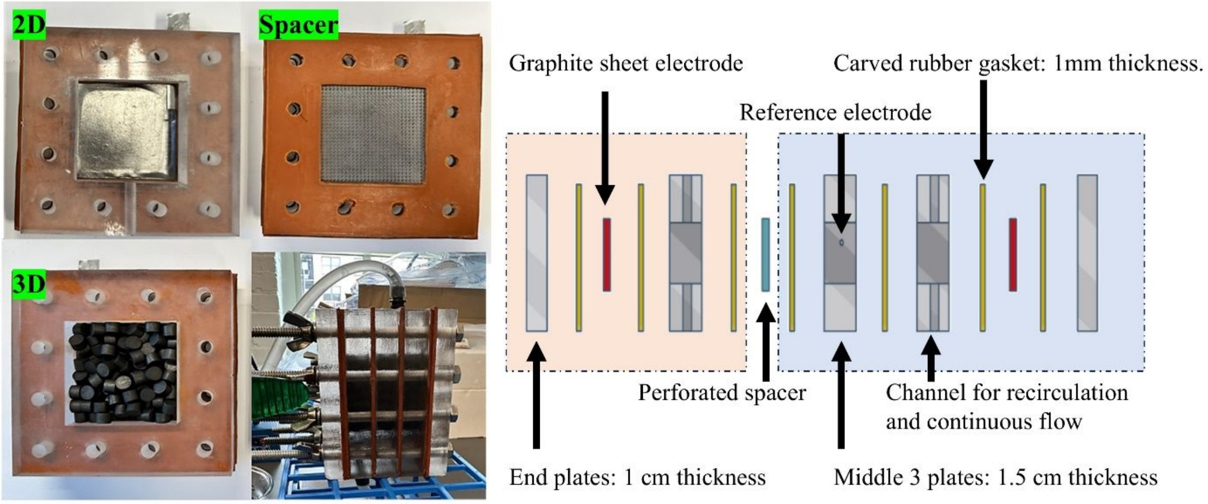

Figure S1. (Left) Pictures of 3DER in section, with a terminal graphite sheet electrode, spacer, and graphite cylinder, and 3DER with complete assembly. (Right) Schematic of the 3DER in cross sections, with orange and blue color blocks indicating the separated chambers. The 5 rubber gaskets in the middle are carved to fit and secure the sheet electrodes and the spacer, which contributes to reactor thickness and provides additional reactor volume.

2. Validation of Se(IV) 6-e reduction pathway in 3DER

Before evaluating the 3DER for Se(IV) reduction performance, we conducted LSV and CV scans under both blank and Se(IV)-spiked conditions to confirm the Se(IV) reduction potential. As shown in Figure S2, the presence of Se(IV) led to two distinct increases in cathodic current, corresponding to the reported 4-electron and 6-electron reduction pathways. The CV results indicate that the desired 6-electron pathway initiates at an applied potential of E_{we}=-0.7 V, which corresponds to E_{cell}=-1.9 V. This confirms that the reduction behavior observed in previous 2D studies can translate to the 3DER system. Accordingly, we selected -1.9 V as the starting potential for evaluating the effect of E_{cell} on Se(IV) reduction.

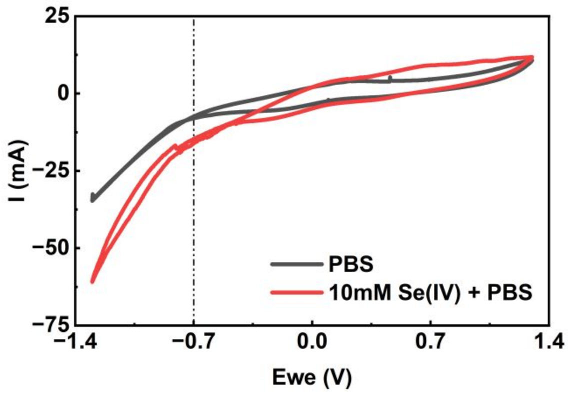

Figure S2. CV scan of 3DER with PBS (Black line) and 10mM Se(IV) spiked PBS (Red line). A high Se(IV) concentration is used to better show the Se(IV) reduction peak position. Significant increase in cathodic current is observed at E_{we}=-0.7V (E_{cell}=-1.9V), indicating Se(IV) 6-e pathway.

3. Validation of cleaning protocol

The cleaning protocol is validated through both experimental and SEM analysis results. We collected treated synthetic water samples with used particle electrodes, and the 12th hour Se(IV) removal is 62.9±4.6%, as compared to the pristine electrodes with 68.1±7.9% removal reported in the manuscript.

Cleaned/Regenerated electrodes were sent out for SEM analysis, and only one of the results came back with Se signal detected. No P, K signals from PBS is detected on the surface either, which proves sufficient surface cleaning with the established cleaning protocol.

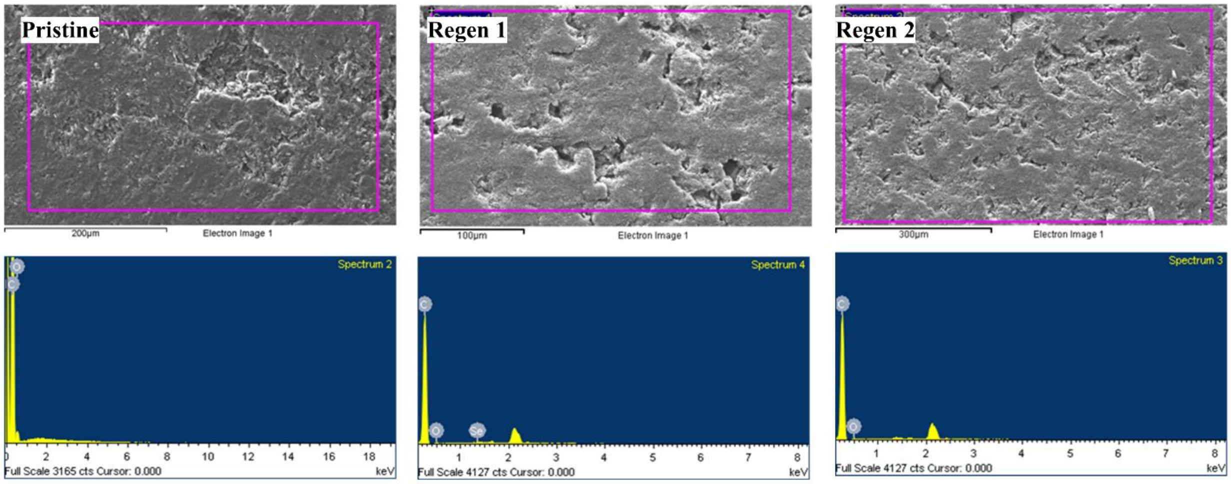

Figure S3. SEM analysis of regenerated particle electrodes.

4. FGD wastewater full spectrum analysis

Full spectrum analysis for FGD wastewater A and B.
Wastewater A and B are collected from different power plants. A is FGD wastewater collected after the plant hydrocyclone process. FGD A sample had high solid content and was filtered again in lab by a 0.45 µm membrane before being used for experiment and sent out for ICP-MS analysis. FGD B is FGD wastewater collected after the sand filtering process, which is used directly in experiments without any alteration.

Table S1. Full ICP-MS analysis for FGD wastewater samples A and B.

	7Li (STDR)	23Na (STDR)	24Mg (STDR)	27Al (STDR)	29Si (STDR)	31P (STDR)	34S (STDR)	35Cl (STDR)	39K (STDR)
	Y (ppb)	Y (ppm)	Y (ppm)	Y (ppb)	Y (ppm)	Y (ppm)	Y (ppm)	Y (ppm)	Y (ppb)
A	528.4±113.2	838.1±72.9	4403.4±510.3	227±17.8	28.9±4.1	30±3.3	1717.9±198.2	28583.2±1740.3	240.2±28.1
B	94.8±30.3	93.4±5.7	1338.4±217.8	295.9±34.7	50±5.9	38.5±7.2	4337.1±621.3	2968.3±361.8	88.2±12.9
	44Ca (STDR)	47Ti (STDR)	51V (STDR)	52Cr (STDR)	54Fe (STDR)	55Mn (STDR)	59Co (STDR)	60Ni (STDR)	65Cu (STDR)
	Y (ppm)	Y (ppb)	Y (ppb)	Y (ppb)	Y (ppb)	Y (ppm)	Y (ppb)	Y (ppb)	Y (ppb)
A	10176.7±298.3	66.2±3.7	186±17.1	7.3±0.8	316.5±15.9	215.8±21.1	612.3±32.5	2492.9±92.7	48.3±0.3
B	774.3±12.7	64.3±2.7	15.9±3.2	3.5±0.3	0	3.3±0.1	6.6±0.1	171.7±6.7	18.8±0.1
	66Zn (STDR)	75As (STDR)	77Se (STDR)	88Sr (STDR)	95Mo (STDR)	107Ag (STDR)	111Cd (STDR)	112Sn (STDR)	138Ba (STDR)
	Y (ppb)	Y (ppb)	Y (ppm)	Y (ppm)	Y (ppb)	Y (ppb)	Y (ppb)	Y (ppb)	Y (ppb)
A	2176±178.5	43±0.8	7.4±0.1	43.8±0.7	34.1±3.4	1.8±0.1	499.6±63.7	478.2±43.8	1470.8±166.5
B	134.8±22.4	4.3±0.1	8.2±0.3	1.9±0.1	46.1±10.5	0	1.1±0.1	2±0.1	39.5±0.7

Treatment train flow diagram:

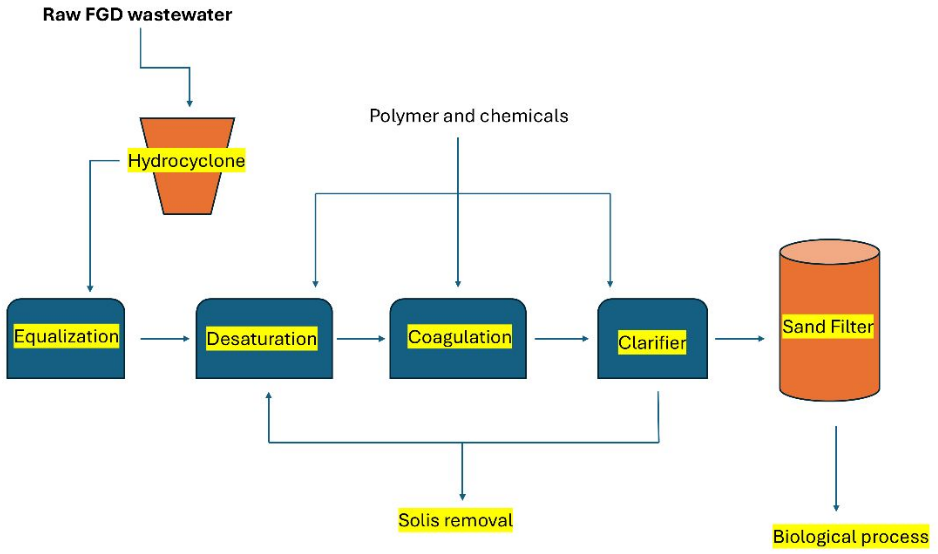

Figure S4. Treatment train flow diagram for FGD wastewater A and B. Recreated based on existing process.

5. E_{we} vs. time recorded data

Due to the high surface area and the consequently high capacitance of carbon-based PEs, the system undergoes a charging process where the E_{we} takes time to reach the equilibrium potential.

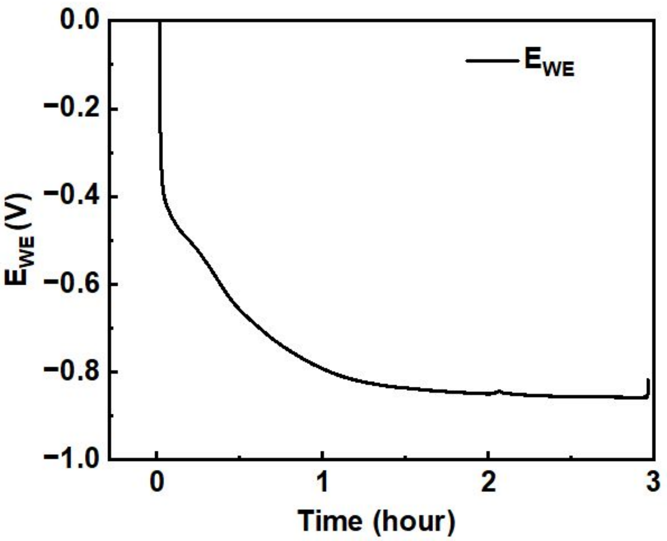

Figure S5. E_{WE} is stabilized around -0.7, -0.8, and -0.9V at E_{cell} of -1.9, -2.1, and -2.3V, respectively, after 1 hour of operation due to the PEs capacitance.

6. Mass titration method for point of zero charge analysis

DI water is adjusted to pH 7 with NaOH and HCl and poured into 50 mL centrifuge tubes to fill. Different weight percent of GAC/GC is added to the solution, and the finished solution is then degassed with N_2 for 30 seconds and sealed. The sealed tubes are shaken for over 12 hours to measure the new pH after equilibrium with GAC/GC.

Table S2. Collected final solution pH from point of zero charge analysis.

WT%	0.5%	1%	1.5%	2%	3%	4%	5%
GAC	10.6	10.7	10.8	10.9	10.8	10.7	10.7
repeat	10.6	10.7	10.8	10.8	10.8	10.7	10.7
GC	7.8	7.8	7.3	6.9	6.6	7.3	8.6
repeat	7.8	7.2	7.0	6.4	6.7	7.9	8.2

The equilibrium pH is the pH_{pzc} of the PE material (GAC pH_{pzc} = 10.7; GC pH_{pzc} = 8.4). When the solution pH is more acidic than the pH_{pzc}, the PEs surface will be positively charged and favor adsorption of anionic species such as Se(IV). Since the GAC has a more basic pH_{pzc}, the GAC PE will have stronger affinity towards Se(IV) in the working solution.

7. Electrochemical impedance analysis for 2DER and 3DER-GC

Table S3. Fitted EIS measurements conducted under varying recirculation rates and E_{cell}.

		ESR			Rc				Rct_Se		
3D	Recirc rate (mL min-1)	R1	Q2	a2	R2	Warburg	Q3	a3	R3	w_max	Cdl (Farad)
**-1.9V**	0.8	4.7	4.77E-05	0.8	4.5	4.5	0.1	0.6	19.8	7655	9.6E-04
	3.3	4.7	5.47E-05	0.8	4.4	4.8	0.1	0.6	18.0	7655	1.0E-03
**-2.1V**	0.8	4.7	9.53E-05	0.7	4.1	3.9	0.0	0.6	21.4	6059	9.9E-04
	3.3	4.7	8.59E-05	0.7	3.9	3.6	0.0	0.5	23.3	6059	8.8E-04
**-2.3V**	0.8	4.7	8.25E-05	0.7	3.8	2.2	0.0	0.5	27.4	6059	7.8E-04
	3.3	4.8	8.68E-05	0.7	3.7	2.4	0.0	0.5	25.2	7655	7.5E-04
2D	Recirc rate (mL min-1)	R1 (ESR)				Warburg	Q3	a3	R3		
**-2.1V**	0.8	18.7	-	-	-	38.3	0.0	0.7	5.8	-	-
	3.3	17.9	-	-	-	13.4	0.0	0.5	22.1	-	-

C_{dl} is calculated as double-layer capacitance using Equation S1:

$C_{dl}=Q_3 * w\_max \wedge (a_3-1)$ (Eq. S1)

8. Schematic and effective potential simulations for 3DER-GC under different chamber ratios

The resistance of the carbon-based particle electrodes soaked in electrolyte was calculated using an equivalent circuit model.[1,2] The electrodes and electrolytes are simplified as resistors connected in parallel and therefore, the total resistance could be calculated using Kirchhoff's Law:

$$\frac{1}{R_{total}} = \frac{1}{R_{electrodes}} + \frac{1}{R_{electrolyte}} \qquad \text{(Eq. S2)}$$

Where R_{total}, $R_{electrodes}$, and $R_{electrolyte}$ represent the total resistance, carbon-based electrode resistance, and electrolyte resistance, respectively. Because the solutions used in this work (PBS solution and FGD wastewater) have significantly lower conductivity (8.7-14.3 mS/cm) than the carbon-based electrodes (~20,000 mS/cm). It is reasonable to assume that R_{total} is close to $R_{electrodes}$ (i.e., $1/R_{electrolyte}$ is close to 0).

Finally, the potential drop on the system was simulated using Ohm/s Law ($V=I*R_{total}$). Where V is the voltage drop (V), I is the current (A) measured with potentiostat, the 'depth' in simulation is the width of the reactor chamber from the end plate to the spacer, and R_{total} is estimated based on a beforehand assumption. The potential drops as 'depth' increases due to the resistance from electrolytes and electrodes.

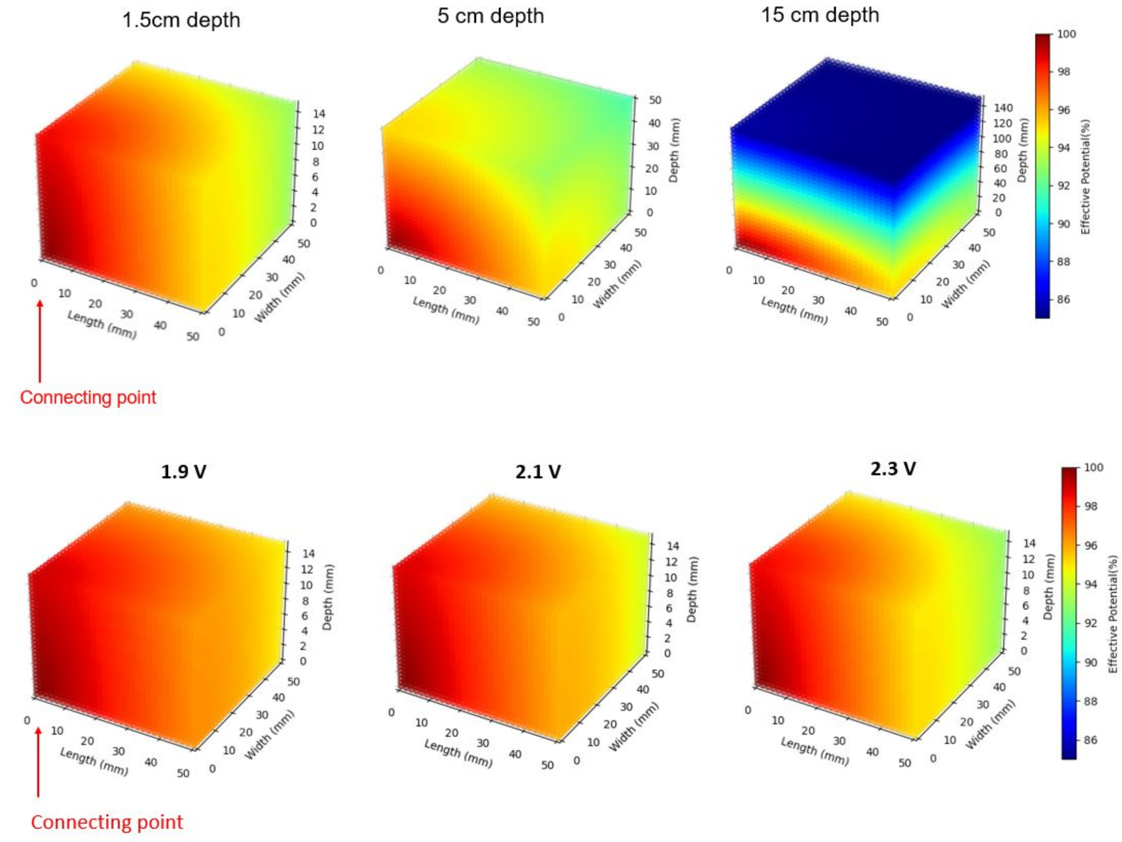

Figure S6. Potential distribution simulation in 3DER-GC.

9. SEM/EDS mapping of PE surface and precipitates

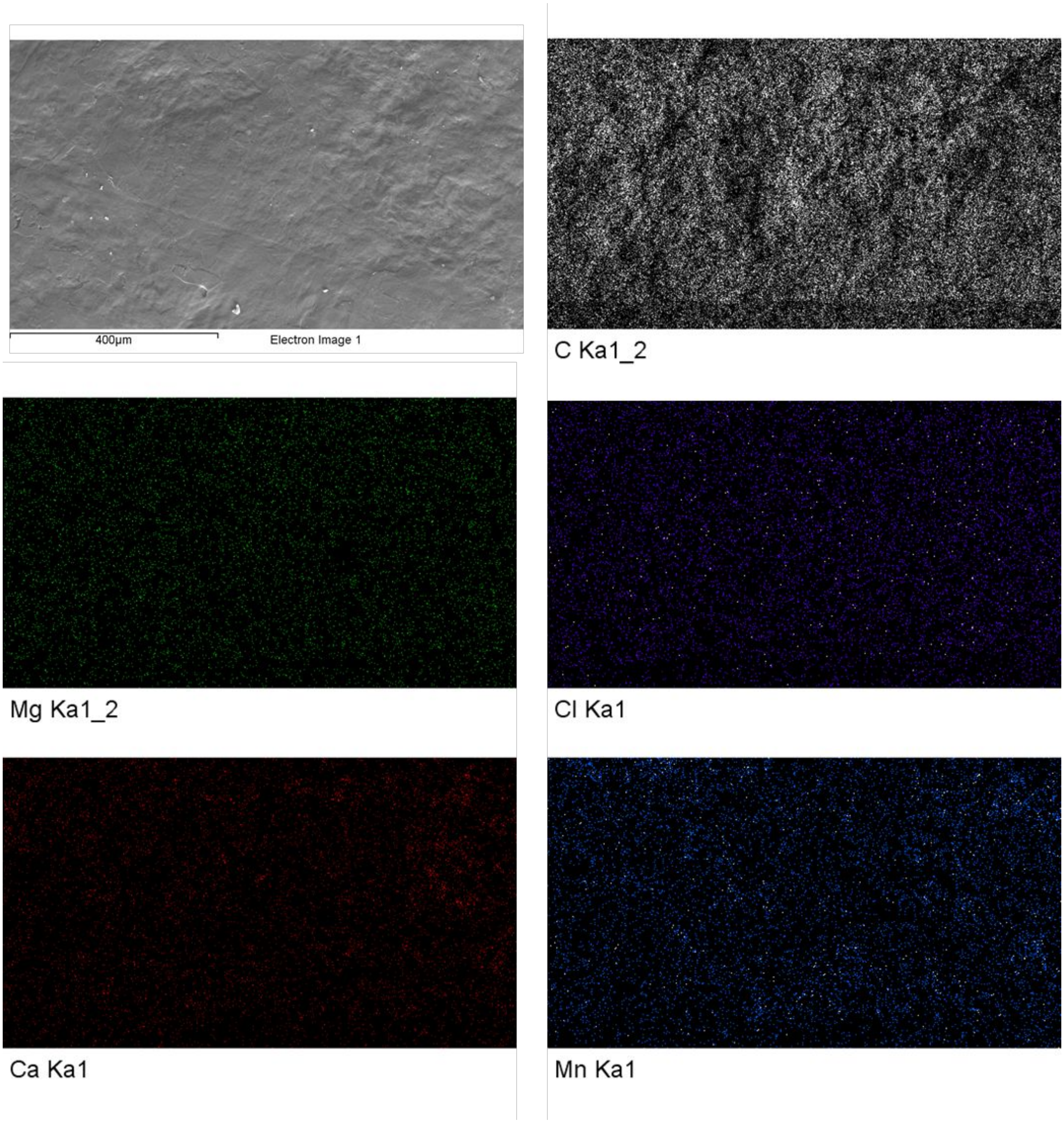

Figure S7. SEM/EDS mapping of GC PEs used in a 12-hour continuous flow experiment with FGD wastewater B. The highlighted dots indicate the presence of the target element, where brighter colors indicate higher concentration.

10. Competing ion experiment in synthetic wastewater

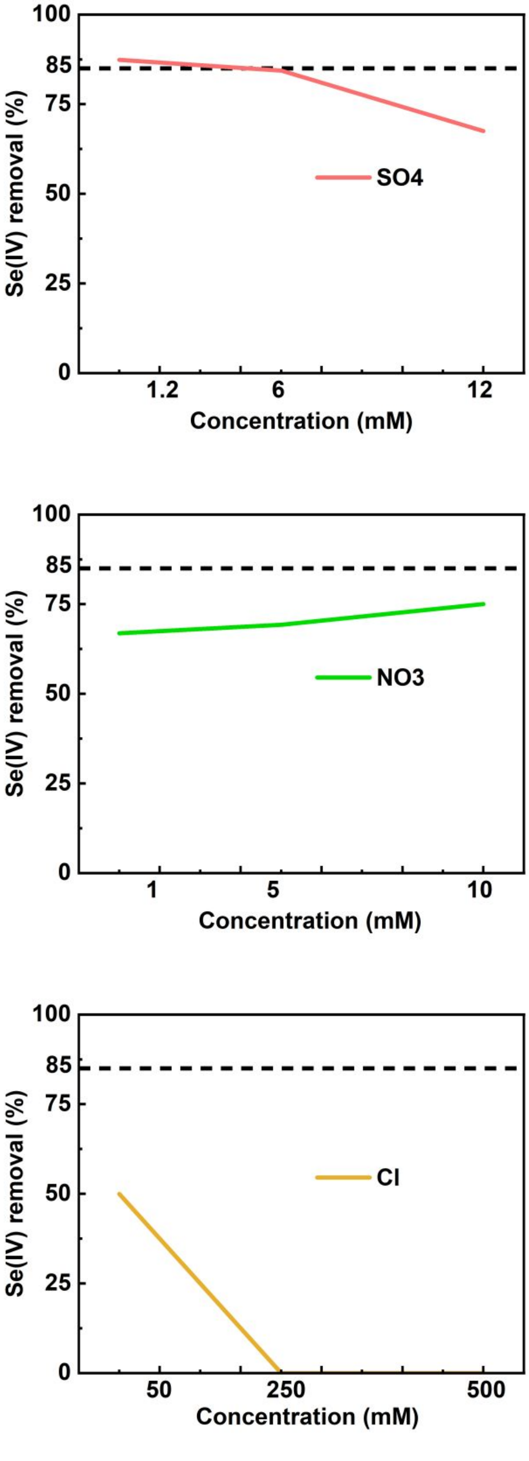

Figure S8. Se removal in synthetic wastewater spiked with competing ions. 100mM PBS + 0.1mM Se(IV) + SO_4/NO_3/Cl.

11. Triplicated experiment data with error bar

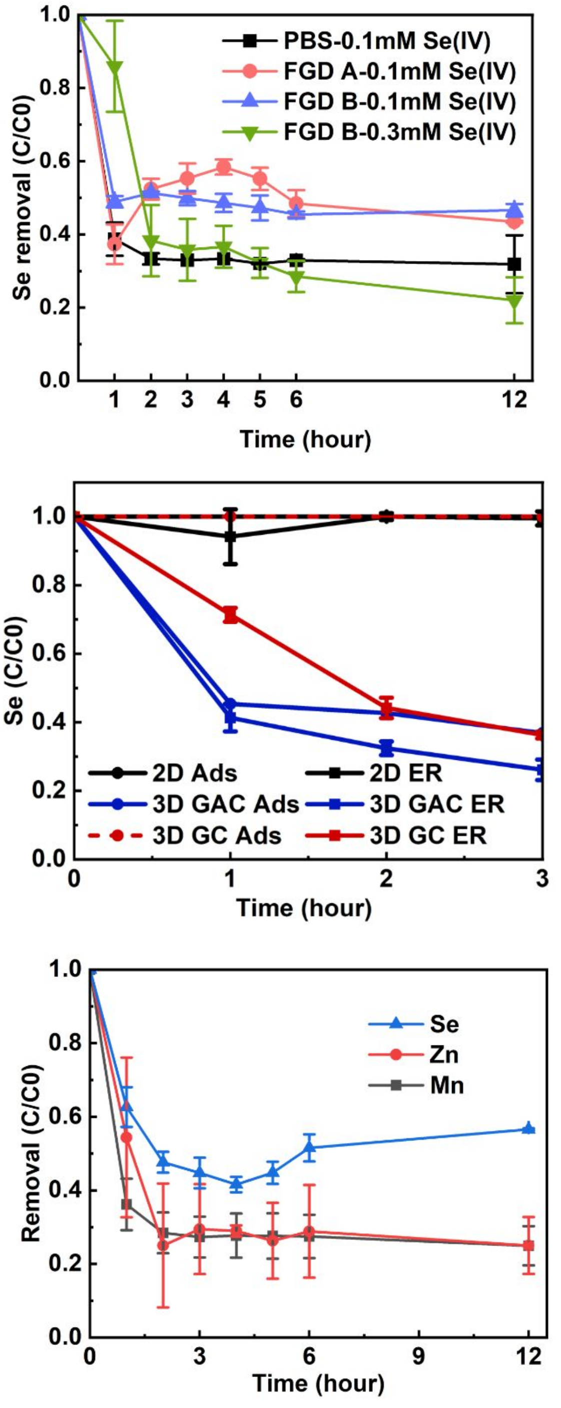

Figure S9. Triplicated experiment data with error bars. From top to bottom, the graphs correspond to Figure 1A, Figure 4A, and Figure 4D.

12. Faradaic efficiency quantification

The theoretical Se removal capacity ($D_{theorectical}$, mg) or the observed Se removal capacity ($D_{observed}$, mg) was determined by:

$$D_{theorectical} = \frac{\Sigma(I \times t)}{n \times F} \qquad \text{(Eq. S3)}$$

$$D_{observed} = C_i \times V_i - C_f \times V_f \qquad \text{(Eq. S4)}$$

where I (A) and t (s) are the current and time profiles recorded by potentiostat, n (6) is the number of electrons required by Se reduction pathway, F is the Faraday constant (96485 C mol^{-1}), C_i and C_f are the initial and final aqueous Se concentrations. V_i and V_f are the initial and final solution volumes, since we are performing a room temperature continuous-flow experiment with minimal solution loss, V_i and V_f are assumed to be equal.

We also quantified the Faradaic efficiency (F, %) of 3DER by:

$$F = \frac{D_{observed}}{D_{theoretical}} \times 100\% \qquad \text{(Eq. S5)}$$

13. Equivalent circuit selection for 3DER EIS

With the addition of graphite particle electrodes in the Nyquist plot, we observed a new semicircle in the Nyquist plot (Fig. S10). This semicircle appears only in the 3DER system, with or without Se(IV). Based on this finding, we identify the semicircle as interparticle resistance. The interparticle resistance is a charge-transfer resistance of polarized particles influenced by their contact-related variables (compactness, compression force, morphology of the particles, conductivity of the external surface, among others), and is not greatly affected by the small changes in polarization here studied.

Since the particles are already charged and Se(IV) reduction did not present any noticeable semicircle, the electrochemical reduction is not significantly resistive in our system. Based on our previous blanks, we decided to allocate the Warburg impedance as the next parameter, in series and after the previous charging process. Meaning after charging the particles, Selenium reduction occurs spontaneously, and it is solely be limited by its mass transport.

We chose the water splitting reaction for the last semicircle due to its higher cathodic potential, implying inherently a more resistive nature, thus being less thermodynamically viable than Se(IV) reduction. In addition, this reaction leads to a loss of the electroactive surface area due to bubbles sticking on the electrode surface and increase in charge transfer impedance.

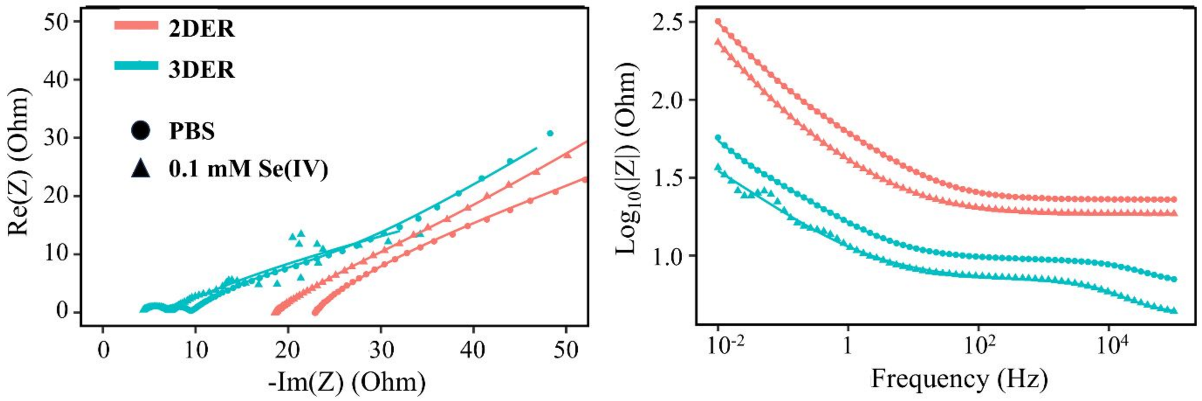

Figure S10. EIS spectrum 2DER and 3DER in PBS and in Se(IV) spiked PBS, experimental condition E_{cell}=-2.1V, recirculation rate = 3.3 mL min^{-1}. (Left) Nyquist plot showing the 3DER has a new semicircle with the introduction of graphite particle electrodes with or without Se(IV), indicating the semicircle is contributed by the interparticle resistance. (Right) Bode plot showing the frequency and system impedance. At the highest frequency of 100kHz, the impedance from mass transfer and charge transfer is negligible as they can't respond to such high frequency, which gives us the equivalent series resistance (ESR). The ESR is the lowest with 3DER and Se(IV) present, while the highest is with 2DER and only PBS.

Reference:

(1) Zhu, X.; Jassby, D. Electroactive Membranes for Water Treatment: Enhanced Treatment Functionalities, Energy Considerations, and Future Challenges. *Acc. Chem. Res.* **2019**, *52* (5), 1177–1186.

(2) Ma, S.; Yang, F.; Chen, X.; Khor, C. M.; Jung, B.; Iddya, A.; Sant, G.; Jassby, D. Removal of As(III) by Electrically Conducting Ultrafiltration Membranes. *Water Res.* **2021**, *204* (February).
